# A network meta-analysis on the comparative efficacy of different dietary approaches on glycaemic control in patients with type 2 diabetes mellitus

**DOI:** 10.1007/s10654-017-0352-x

**Published:** 2018-01-04

**Authors:** Lukas Schwingshackl, Anna Chaimani, Georg Hoffmann, Carolina Schwedhelm, Heiner Boeing

**Affiliations:** 1 Department of Epidemiology, German Institute of Human Nutrition Potsdam-Rehbruecke (DIfE), Arthur-Scheunert-Allee 114-116, 14558 Nuthetal, Germany; 2 Paris Descartes University, Paris, France; 3 INSERM, UMR1153 Epidemiology and Statistics, Sorbonne Paris Cité Research Center (CRESS), METHODS Team, Paris, France; 4 Cochrane France, Paris, France; 5 Department of Nutritional Sciences, University of Vienna, Althanstraße 14, 1090 Vienna, Austria

**Keywords:** Systematic review, Diet, Type 2 diabetes mellitus, Network meta-analysis, Evidence synthesis

## Abstract

The aim of the present study is to assess the comparative efficacy of different dietary approaches on glycaemic control in patients with type 2 diabetes mellitus using a systematic review of the literature. Electronic and hand searches were performed until July 2017. The inclusion criteria were defined as follows: (1) randomized trial with a dietary approach; (2) adults with type 2 diabetes mellitus; (3) outcome either HbA1c (%) and/or fasting glucose (mmol/l); (4) minimum intervention period of 12 weeks. For each outcome measure, random effects network meta-analysis was performed in order to determine the pooled effect of each intervention relative to each of the other interventions. A total of 56 trials comparing nine dietary approaches (low-fat, Vegetarian, Mediterranean, high-protein, moderate-carbohydrate, low-carbohydrate, control, low GI/GL, Palaeolithic) enrolling 4937 participants were included. For reducing HbA1c, the low-carbohydrate diet was ranked as the best dietary approach (SUCRA: 84%), followed by the Mediterranean diet (80%) and Palaeolithic diet (76%) compared to a control diet. For reducing fasting glucose, the Mediterranean diet (88%) was ranked as the best approach, followed by Palaeolithic diet (71%) and Vegetarian diet (63%). The network analysis also revealed that all dietary approaches significantly reduce HbA1c (− 0.82 to − 0.47% reduction) and fasting glucose (− 1.61 to − 1.00 mmol/l reduction) compared to a control diet. According to the network meta-analysis the Mediterranean diet is the most effective and efficacious dietary approach to improve glycaemic control in type 2 diabetes patients.

**Electronic supplementary material:**

The online version of this article (10.1007/s10654-017-0352-x) contains supplementary material, which is available to authorized users.

## Background

According to the most recent data by the International Diabetes Federation and the World Health Organization, type 2 diabetes (T2D) represents one of the most important health problems, causing enormous costs, with an estimated prevalence of 350–400 million cases worldwide [1, 2].

To prevent onset of T2D, high-quality diets have been recognized to play a critical role [3–5]. Nutrition therapy plays an integral role in the management of T2D, particularly after initial clinical diagnosis, in order to reduce or delay diabetes associated complications. One major approach is the loss of weight by a hypocaloric diet [6]. However, there is limited evidence on the optimal dietary approaches to control hyperglycaemia in T2D patients [7] and uncertainty regarding the optimal proportion of energy coming from carbohydrates, protein, and fat for patients with T2D [8].

Meta-analyses showed that some dietary approaches such as a low-carbohydrate, low-glycaemic index/load, high protein-, Vegetarian-, and Mediterranean dietary approaches were effective in reducing HbA1c [9, 10]. Nevertheless, other meta-analyses reported conflicting results [7, 11, 12].

**Fig. 1:**
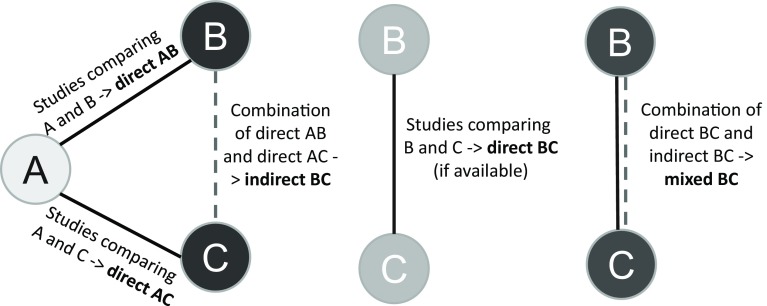
Example of direct, indirect and mixed relative effects in a hypothetical triangle comparing three interventions

One of the most important questions that remain to be answered is which dietary approach offers the greatest benefits. For answering this question, a promising method is network meta-analysis (NMA), which is an extension of pairwise meta-analysis that enables a simultaneous comparison of multiple interventions. NMA combines direct (i.e., from trials comparing directly two interventions) and indirect (i.e., from a connected root via one more intermediate comparators) evidence in a network of trials (Fig. 1). In this way, it enables inference about every possible comparison between a pair of intervention in the network even when some comparisons have never been evaluated in a trial. A fundamental assumption of NMA, often called the transitivity assumption, is that trials comparing different sets of interventions (e.g., AB and AC trials) should be similar enough in all characteristics that may affect the outcome. For more details on the methodology of NMA we directed the readers to relevant tutorials [13–15].

To our knowledge, no study has been conducted that compared simultaneously different dietary approaches in the management of T2D. Therefore, our aim is to compare the efficacy of different dietary approaches in clinical trials on glycaemic control in patients with T2D using the novel method of NMA.

## Methods

The review was registered in PROSPERO International Prospective Register of Systematic Reviews https://www.crd.york.ac.uk/prospero/display_record.php?RecordID=47464 and our strategy for the systematic review and NMA was pre-defined in a published protocol [16]. The present systematic review was planned, conducted, and reported in adherence to standards of quality for reporting systematic reviews and NMA [17, 18].

### Search strategy

The literature search was performed using the electronic databases PubMed, Cochrane Central Register of Controlled Trials (CENTRAL), and Google Scholar until July 2017 with no restriction to language and calendar date using a pre-defined search strategy (Supplementary Appendix S1).

Furthermore, systematic reviews, and the reference lists from the retrieved articles were screened to search for additional relevant studies. Searches were conducted by two authors with disagreements being resolved by involvement of another reviewer.

### Eligibility criteria

Studies were included in the review if they met all of the following criteria:

(i) Randomized comparison study design (parallel or cross-over) between different dietary approaches (energy restricted diets, iso-caloric, or ad libitum diets):
  - Low carbohydrate (LC) diet (< 25% carbohydrates of total energy intake; high intake of animal and/or plant protein; often high intake of fat) [19];
  - Moderate-carbohydrate diet (25–45% carbohydrates of total energy intake; 10–20% protein intake) [19];
  - High protein (HP) diet (> 20% protein intake of total energy intake; high intake of animal and/or plant protein; < 35% fat) [20];
  - Low fat (LF) diet (< 30% fat of total energy intake; high intake of cereals & grains; 10–15% protein intake) [7, 19];
  - Low glycaemic index/load (LGI/GL) diet [21, 22];
  - Vegetarian/Vegan diet (no meat and fish/no animal products) [23];
  - Mediterranean dietary pattern: fruit, vegetables, olive oil, legumes, cereals, fish, and moderate intake of red wine during meals [5, 24–28];
  - Palaeolithic diet [29];
  - Control diet: no intervention or minimal intervention [30];
(ii) Minimum intervention period of 12 weeks;
(iii) Patients with a mean age ≥ 18 years, following the diagnosis criteria of the American Diabetes Association or according to the internationally recognized standards for patients with T2D [31];
(iv) The primary outcome is glycosylated haemoglobin HbA1c (%) and the secondary outcome was defined fasting glucose (mmol/l).

The following studies were excluded:

(i) Randomized trials including pregnant women, children, and adolescents, patients with abnormal glucose metabolism;
(ii) Intervention studies solely based on dietary supplements or single foods;
(iii) Intervention studies using dietary supplements as placebo;
(iv) Studies with an exercise/medication [32, 33] co-intervention that was not applied in all the intervention/control groups;
(v) Interventions based on very low energy diets (i.e., < 600 kcal/day).

### Data extraction

After determination of the study selection, two reviewers extracted the following characteristics: name of first author, year of publication, study origin (country), study design (RCT: parallel or cross-over), sample size, mean baseline age, mean baseline BMI, mean baseline HbA1c, study duration, sex, description of the different dietary intervention arms, specification of the control group, type of diet (energy restricted, ad libitum, iso-caloric), drop outs, presence of comorbidities, hypoglycaemic drugs, antihypertensive medication, lipid lowering medication. Outcome data include: post-intervention values with corresponding standard deviations for glycosylated haemoglobin and fasting plasma glucose.

### Risk of bias assessment

Full copies of the studies were assessed by two authors for methodological quality using the risk of bias assessment tool from the Cochrane Collaboration [34]. The following sources of bias were assessed: selection bias (random sequence generation and allocation concealment), performance bias (blinding of participants and personnel), attrition bias (incomplete outcome data), and reporting bias (selective reporting).

Studies were classified as being at low risk of bias (if at least three out of a maximum of five items were rated as low risk; and maximum one item rated with a high risk of bias), high risk of bias (if at least two out of a maximum of five items were rated as high risk), and moderate/unclear risk (all other studies) using the risk of bias assessment tool from the Cochrane Collaboration.

### Dealing with missing data

We contacted authors to receive missing outcome data (3 authors sent additional data, see acknowledgements). If the post-intervention values with the corresponding standard deviations were not available, the change scores with the corresponding standard deviations were used, according to the guidelines of the Cochrane Handbook [35].

## Evaluation of synthesis assumptions

### Data synthesis

#### Description of the available data

We present for all included trials study and population characteristics describing the available data and important variables (e.g., age, length of follow-up, outcome relevant baseline risk factors, etc.). We illustrate the available direct comparisons between different dietary interventions and control group using a network diagram for each outcome [36]. The size of the nodes is proportional to the sample size of each dietary intervention and the thickness of the lines proportional to the number of studies available.

#### Assessment of transitivity

Transitivity is the fundamental assumption of indirect comparisons and NMA, and its violation threatens the validity of the findings obtained from a network of studies. To evaluate the assumption of transitivity we compared the distribution of the potential effect modifiers across the available direct comparisons. We considered the following effect modifiers: body weight, duration of diabetes, mean baseline age, and study duration.

#### Statistical analysis

For each outcome measure of interest, we performed random effects NMA in order to determine the pooled relative effect of each dietary intervention against every other intervention in terms of the post-intervention values. NMA was used to synthesize the direct and indirect effects. The method of NMA is an extension of the standard pairwise meta-analysis that enables a simultaneous comparison of multiple interventions, forming a connected network while preserving the internal randomization of individual trials. We ran random effects NMA for each outcome to estimate all possible pairwise relative effects and to obtain a clinically meaningful relative ranking of the different dietary interventions. We present the summary mean differences with their 95% CI in a league table. We estimated the relative ranking of the different diets for each outcome using the distribution of the ranking probabilities and the surface under the cumulative ranking curves (SUCRA) [37]. For each outcome we assumed a common network-specific heterogeneity parameter and estimated the predictive intervals to assess how much this heterogeneity affects the relative effects with respect to the additional uncertainty anticipated in future studies [38]. We fitted all analyses described in a frequentist framework using Stata [39] (*network* package [40]) and produced presentation tools with the *network graphs* package [41].

#### Assessment of inconsistency

To evaluate the presence of statistical inconsistency (i.e., disagreement between the different sources of evidence) in the data, we employed both local and global approaches [42]. Specifically, we used the loop-specific approach [43] to detect loops of evidence that might present important inconsistency as well as the side-splitting approach [44] to detect comparisons for which direct estimates disagree with indirect evidence from the entire network. Global methods investigate the presence of inconsistency jointly from all possible sources in the entire network simultaneously. For this purpose, we used the design-by-treatment interaction model [45, 46].

#### Subgroup and sensitivity analyses

For comparability reasons, we performed subgroup analyses in accordance to previous pairwise meta-analyses investigating the effects of dietary interventions, by taking into account study duration (≥ 12 vs. < 12 months) [19, 47], sample size (≥ 100 vs. < 100) [48], and age (≥ 60 vs. < 60 years) [48]. We also conducted sensitivity analyses by analysing only studies considered being at low risk of bias, and by excluding risk of bias trials. We ran also a meta-regression analysis to investigate the association between the primary outcome (HbA1c) and mean differences in weight change.

#### Small study effects and publication bias

We drew inference on the risk for publication bias based primarily on non-statistical considerations; hence by considering how likely it is that studies may have been conducted but not published based on the expertise of the investigators in the field. We also produced the comparison-adjusted funnel plot [36] and fit a network meta-regression model to assess the magnitude of funnel plot asymmetry for the primary outcome.

#### Credibility of the evidence

To make inferences about the credibility of evidence from the NMA we used the GRADE system extended for NMA following the approach suggested by Salanti et al. (see the Supplementary Appendix S2 for details) [42].

## Results

Out of 3852 records identified by the literature search, 115 full text articles were assessed in detail as they reported on one or more of dietary approaches and T2D in the title/abstract (Supplementary Figure S1). Of these, 59 were excluded, with the reasons for exclusion summarized in Supplementary Table S1.

Overall, 56 trials [29, 49–103] met the eligibility criteria and provided sufficient data to be included in the meta-analysis. The included studies were published between 1978 and 2016 and had enrolled a total of 4937 T2D patients. Eighteen trials were conducted in North America, 14 trials in Europe, 8 trials in Asia, and 16 trials in Australia and New Zealand. The study duration ranged between 3 and 48 months; the patients’ mean age was between 44 and 67 years, and their BMI between 25 (Asian population) and 43 kg/m^2^. The general and specific study characteristics are summarized in Supplementary Table S2 and S3.

Twenty-one trials were judged to be low risk–, seven trials to be high risk of bias, and 28 trials were classified as moderate/unclear risk of bias studies. With regard to the specific items of the risk of bias assessment tool by the Cochrane Collaboration, 56% of the included studies indicate a low risk of bias for random-sequence generation, 23% for allocation concealment, 0% for blinding, 63% for incomplete data outcome, and 79% for selective reporting (Supplementary Figure S2).

The studies applied heterogeneous definitions for the different intervention diets. The fat intake varied across the different LF trials by ~ 10–15% of total energy intake, and also the intervention protocols varied among the trials (i.e., group meeting, dietary counselling, and intensity). Moreover, hypocaloric, iso-caloric, and ad libitum diets were included in the NMA. Moreover the definition of a control diet showed some difference across the included trials. Four out of the ten trials were based on “no intervention”, whereas the other six trials were based on minimal intervention (standard dietary advice). We thus had to harmonize the single studies and formed classes of dietary approaches.

**Fig. 2:**
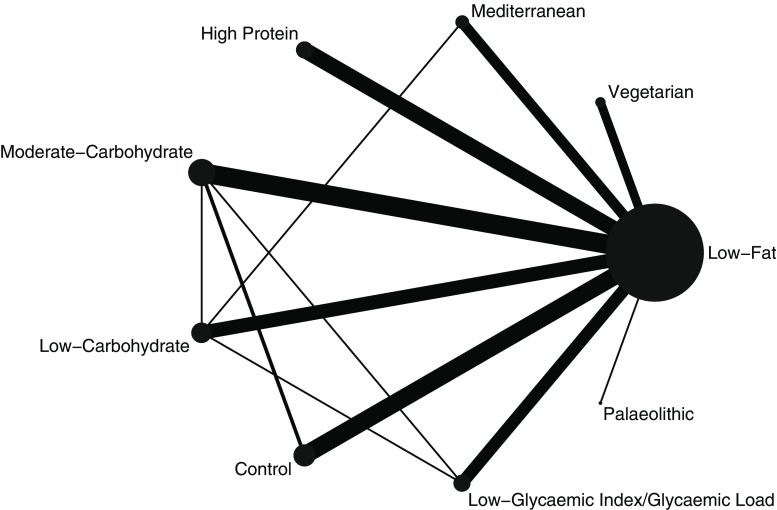
Network diagram for HbA1c: The size of the nodes is proportional to the total number of participants allocated to each dietary approach and the thickness of the lines proportional to the number of studies evaluating each direct comparison

Figure 2 shows the network diagrams of direct comparison for HbA1c with the number of studies reflected by the size of the edges, and the number of patients reflected by the size of the nodes. The highest number of trials include moderate-carbohydrate diet compared to LF diets [68–80] (n = 13), LF diet compared to control diets [72, 73, 88–95] (n = 10), HP diet compared to LF diets [60–67] (n = 8), and LC diet compared to LF diets [57, 81–87] (n = 8).

NMA simultaneously analyse both direct comparisons of interventions within trials and indirect comparisons across trials based on a common comparator. Since none of the studies have compared B (Vegetarian) and C (Mediterranean), but each has been compared with a common comparator A (LF), then we assume an indirect comparison of B and C on the direct comparison of B and A and the direct comparison of C and A [104]. Table 1 shows the percentage of statistical contribution coming from direct and indirect comparisons for each dietary approach compared to each other. It was shown that most of the contribution to the study effects came from indirect comparisons. Direct comparisons dominated the comparisons of Vegetarian/Mediterranean/HP/moderate-carbohydrate/LC/LGI/GL/Palaeolithic/control diet with a LF diet for both outcomes. In general, there are no important differences in the examined effect modifiers across comparisons apart from the duration of diabetes which does not seem to be distributed similarly across the different comparisons. For some comparisons such as LC versus LGI/GL, LC versus moderate carbohydrate, LGI/GL versus moderate carbohydrate, and Palaeolithic versus LF, we do not have enough studies and we could not test transitivity appropriately (Supplementary Figure S3–6).

**Table 1:**
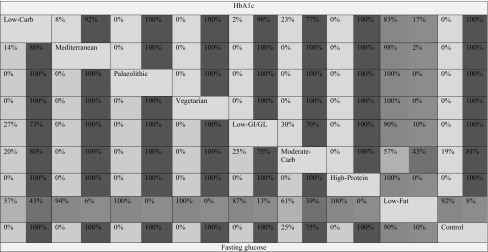
Percentage contribution of each direct estimate derived from direct (blue) and indirect (red) comparisons (the colour corresponds to the percentage of contribution)

The values above the dietary approaches correspond to the percentage contribution of direct and indirect comparisons between the row and columns for HbA1c (e.g., the percentage contribution of direct comparisons for HbA1c between Low-Carb and Low-Fat diet is 83%, and 17% for the indirect comparisons). The values below the dietary approaches correspond to the percentage contribution of direct and indirect comparisons between the column and the rows for fasting glucose (e.g., the percentage contribution of direct comparisons for fasting glucose between Low-Carb and Low-Fat diet is 57%, and 43% for the indirect comparisons). GI/GL, glycaemic index/load. (Color table online)

The effect size estimates for the comparison of every dietary approach compared with each other dietary approach on HbA1c and fasting glucose outcomes are given in Table 2. All dietary approaches were more effective in reducing HbA1c (− 0.82 to − 0.47% reduction) and fasting glucose (− 1.61 to − 1.00 mmol/l reduction) compared to a control diet. The Mediterranean (MD: − 0.32, 95% − 0.53, − 0.11) and the LC diet (MD: − 0.35, 95% − 0.56, − 0.14) were more effective in reducing HbA1c compared to a LF diet. Moreover, the LC diet was also more effective in HbA1c reduction compared to a HP diet (MD: − 0.33, 95% − 0.61, − 0.05). The Mediterranean diet was more effective in reducing fasting glucose compared to a LF- (MD: − 0.61 mmol/l, 95% − 1.03, − 0.20) and LGI/GL diet (MD: − 0.59 mmol/l, 95% − 1.13, − 0.04) (Supplementary Figure S7 and S8, Table 2). In addition, the LGI/GL diet was associated with a trend for a reduction in HbA1c compared to the LF diet (MD: − 0.16, 95% − 0.31, − 0.00). The LC diet had the highest SUCRA value (84%), followed by the Mediterranean diet (80%), and Palaeolithic diet (76%) for HbA1c, whereas the Mediterranean diet (88%) had the highest SUCRA value for fasting glucose, followed by Palaeolithic diet (71%) and Vegetarian diet (63%) (Table 2). The rankograms did not imply the presence of important uncertainty in ranking for HbA1c; more uncertain appeared to be the relative ranking for fasting glucose though (Supplementary Figure S9 and S10).

**Table 2:**
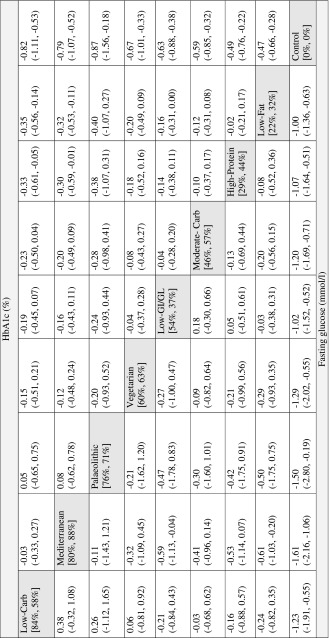
League table showing the results of the network meta-analysis comparing the effects (mean difference: MD) of all dietary approaches and 95% confidence intervals (95% CI)

The values above the dietary approaches correspond to the MD and 95% CI in HbA1c (%) between the row and columns (e.g., the MD in HAb1c between Low-Carb and Low-Fat diet is −0.35%). The value below the dietary approaches correspond to the MD in fasting glucose (mmol/l) between the column and the row (e.g., the MD fasting glucose between Low-Carb and Low-Fat diet is −0.24 mmol/l). The values in square brackets represent the SUCRA for HbA1c and fasting glucose (e.g., the LC diet was ranked as the best dietary approach for reducing HbA1c, SUCRA: 84%; the Mediterranean diet was ranked as the best dietary approach for reducing fasting glucose, SUCRA: 88%). GI/GL, glycaemic index/load

The side-splitting approach suggested important inconsistency for HbA1c in the comparisons of LF versus LC, LF versus control diet, and moderate-carbohydrate versus LC diet (Supplementary Table S4 and S5). For fasting glucose, significant inconsistency was observed for LF versus LC diet and moderate-carbohydrate versus LC diet. The loop-specific approach showed important inconsistency in the loop formed by the aforementioned diets for both outcomes (Supplementary Figure S11 and S12). The design-by-treatment model showed also significant inconsistency for HbA1c (*p* = 0.03), but not for FG (*p* = 0.32).

This apparent inconsistency might reflect the low contribution of direct comparisons to the total estimate. The important inconsistency in the loop for LF versus moderate-carbohydrate and LC might be explained by several differences across LF dietary approaches (hypocaloric if compared to a control diet; often iso-caloric if compared to other dietary approaches), differences in ratio of fat to carbohydrate intake, and differences in fatty acid composition among moderate carbohydrate approaches and LC dietary approaches (larger weight loss compared to other interventions).

In the subgroup analyses for study duration, sample size, and age we could show that LC diets were more effective in reducing HbA1c in the shorter-term (< 12 months), in smaller size studies, and including patients ≥ 60 years. Mediterranean, moderate-carbohydrate and LGI/GL, HP, and LF diets were more effective in reducing HbA1c in the longer-term, in larger size studies, and in studies including patients < 60 years (Supplementary Table S6–11). Although the power was very small for several comparisons, these characteristics may partly explain the presence of inconsistency. Furthermore, in the low-risk of bias sensitivity analysis the results of the primary analysis were generally confirmed. Hence, both the Mediterranean diet and the LC diet were more effective to decrease HbA1c compared to a LF diet, whereas the results for FG were not significant (Supplementary Table S12). All the results of the main analysis were confirmed in the sensitivity analysis excluding high risk of bias trials (Supplementary Table S13). In univariate meta-regression analysis we could show that mean reduction in HbA1c was significantly (*p* = 0.04) related to mean difference in weight change between dietary approaches (Supplementary Figure S11).

The comparison-adjusted funnel plots for both outcomes appear slightly asymmetric when LF dietary approaches were compared to all other dietary approaches. However, the network meta-regression model that accounted for differences in study variance did not yield a statistically significant coefficient (Supplementary Figure S14 and S15).

The credibility of evidence was rated very low for the comparisons Mediterranean versus LF; LC versus LF; LGI versus LF, moderate-carbohydrate versus LC, Mediterranean versus HP, and LC versus HP. The very low credibility was driven by significant inconsistency. For the other comparisons the credibility of evidence was rated low, and for three comparisons the quality of evidence was rated moderate (LF vs. Palaeolithic, Mediterranean vs. Palaeolithic, LGI/GL vs. Palaeolithic) (Supplementary Figure S16, Supplementary Appendix S2).

## Discussion

By applying NMA, we ranked 9 different dietary approaches (Vegetarian, Mediterranean, HP, moderate-carbohydrate, LC, LGI/GL, Palaeolithic, LF and control diet) regarding their comparative efficacy for glycaemic control in patients with T2D. The ranking according to SUCRA showed the highest value for the LC diet, followed by the Mediterranean diet, and Palaeolithic diet for HbA1c, whereas the Mediterranean diet had the highest SUCRA value for fasting glucose, followed by Palaeolithic diet and Vegetarian diet. However, the credibility of evidence was rated very low for the LC, as well as for some comparison with the Mediterranean diet. The NMA also revealed that all dietary approaches significantly reduce HbA1c (− 0.47 to − 0.82% reduction) and fasting glucose (− 1.00 to − 1.61 mmol/l reduction) compared to a control diet.

In line with our observations, pairwise meta-analyses have shown that LC diets were more effective in HbA1c and body weight reduction in the short-term compared to other diets, whereas no superiority was observed in the long-term [105, 106]. Weight loss as an important effect modifier for HbA1c and fasting glucose reduction may potentially explain the observed inconsistency between LC and the other dietary approaches. Despite the moderate quality of evidence grading in the NMA, the findings for Palaeolithic diet should be interpreted with caution since only one trial was available. Finally, it is important to note that LC diets were more effective in reducing HbA1c in patients ≥ 60 years, whereas the Mediterranean, moderate-carbohydrate, LGI/GL, HP, and LF diets were more effective in HbA1c reduction in patients < 60 years, compared to patients ≥ 60 years. Irrespective of the age of the study participants, HbA1c reductions have been reported to be of similar degree following either LC or LF dietary regimens [87]. In contrast, other studies demonstrated stronger decreases in HbA1c in individuals subjected to a LC approach [73]. It remains speculative whether these differences might be due to an age-dependency of LC effectiveness as shown in the present subgroup analysis. Given the fact that various authorities have proposed specific guidelines for glycemic control in older adults [107, 108] to minimize the risk of hypoglycemia, these observations need to be confirmed by larger RCTs mainly in patients ≥ 60 years.

In the past, with traditional pairwise meta-analysis, Ajala and co-workers compared various diets modifying macronutrient intake on glycaemic control and weight loss in patients with T2D [10]. In 2003, Brand-Miller et al. [109] could show a beneficial effect specifically of LGI foods as compared to regular or high GI diets on HbA1c and fructosamine in subjects with type 1 or type 2 diabetes, however, this study included mostly randomized trials with a duration time of less than 12 weeks, whereas we included only trials with a minimum intervention period of 12 weeks. The duration of time is an important factor in dietary- and overall lifestyle intervention trials, since participants adherence declines over time, and improvements in risk factors are often larger in the short term, compared to the longer term [57, 110].

Comparable effects were shown for carbohydrate-restricted diets by Kirk et al. [111]. In a meta-analysis of RCTs by Huo et al. [112], a Mediterranean diet did result in significantly more pronounced decreases in parameters of glycaemic control and weight loss as compared to control diets. In addition, Dong et al. [113] observed improvements in HbA1c but not in fasting plasma glucose following a meta-analytical synthesis of data from randomized trials comparing HP with low-protein diets. In another meta-analysis, Yokoyama et al. [9] demonstrated favourable effects of Vegetarian diets on glycaemic control in patients with T2D. The results of our NMA extend the current knowledge from previous pairwise meta-analyses, since we were the first to rank 9 different dietary approaches regarding their comparative efficacy by analysing simultaneously both direct and indirect effects. We could show that a plant-based diet such as the Mediterranean diet is the most effective dietary approach to improve glycaemic control in T2D patients. This will affect evidence-based decision-making with respect to dietary regimens by providing a reliable basis for dietary recommendations in the management of T2D.

With respect to mechanisms of action, the effects of LC diets, Mediterranean diets or Palaeolithic diets on HbA1c might be mediated by their higher amounts of food groups such as fruits, vegetables, or whole grains providing antioxidants or fibre, known to improve insulin sensitivity or to directly inhibit production of advanced glycosylated end products [114–116]. The additional benefit of a Mediterranean diet on fasting plasma glucose might be exerted via dietary polyphenols (e.g., flavonoids, phenolic acids, resveratrol, lignans) provided by key components of the Mediterranean diet such as olive oil, nuts, red wine, legumes, fruits, and vegetables [48, 117–119]. Moreover, the meta-regression analysis showed that HbA1c reduction was significantly related to mean differences in weight change, indicating that weight loss is another important mechanism to improve glycaemic control.

Both HbA1c and fasting plasma glucose are considered to be clinical tools for the assessment of glycaemic control. However, these parameters might not accurately determine short-term fluctuations in glycaemia within a day or long-term variations within several months. Glycaemic variability is supposed to be an independent predictor of diabetic complications [120].

Optimal control of glycaemic parameters in T2D subjects is an essential step to reduce the risk of long-term health damages associated with the disease. According to the Asian Pacific Study, attenuations in fasting glucose levels of 1 mmol/l are associated with a 23% lower risk of CVD [121]. Moreover, the authors of the United Kingdom Prevention Study considered hyperglycaemia to be a more relevant predictor of coronary events in the course of T2D when compared with increased insulin levels [122]. In a retrospective study by Currie et al. [123] investigating 47,970 patients with T2D, HbA1c values higher than 6.5% were associated with an increased mortality rate. In the EPIC-Norfolk study, an increase in HbA1c of 1 percentage point was associated with a 20–30% increase in mortality or in risk of cardiovascular events [124]. Likewise, an HbA1c increase of 1 percentage point was associated with a relative risk for death from any cause of 1.24 in men and 1.28 in women [124]. This underlines the validity of HbA1c and fasting plasma glucose in monitoring the management of T2D.

### Strength and limitations

This systematic review includes the application of novel NMA methods, which simultaneously combine direct and indirect evidence. Additional strengths are the high number of included trials, the comprehensive literature search, the a priori published systematic review protocol, identification of inconsistency, and the credibility of evidence assessment.

A limitation of this review lies within the number and qualities of the studies available. Overall, 7 of 56 trials were at high risk of bias mostly due to lack of allocation concealment, and blinding. However, the sensitivity analysis excluding the high risk of bias trials confirmed all the results of main NMA. Another important limitation is that analyses were based on the original intended randomized design, not by adherence to the actual dietary approach and/or macronutrient composition and caloric intake consumed. This means that although patients were randomized to various diets or controls, details on their actual adherence to the dietary program were not accounted for in the analyses. The heterogeneous definition for the different dietary approaches and the overlap between some dietary approaches is another limitation. In some cases a LGI/GL or a HP diet would also fulfil the criteria of a LF diet, whereas on the contrary a LF would never fulfil the criteria of other dietary approaches. The observed statistical inconsistency, which was also reflected in the GRADE assessments, is another important limitation of the ranking and lowers the confidence in the effect estimates being used in the analysis. As shown in the subgroup analyses we observed significant differences between LC compared to other dietary approaches for study duration, sample size, and patients’ age.

## Conclusion

According to the NMA, the Mediterranean diet seems to be the most effective and efficacious dietary approach to improve glycaemic control in T2D patients. These findings need to be seen under the light of very low to moderate credibility of evidence. However, the findings could nevertheless influence dietary recommendations in the management of T2D.

## Electronic supplementary material

Below is the link to the electronic supplementary material.

Supplementary material 1 (PDF 384 kb)
