# Supplementary material for: A network meta-analysis on the comparative efficacy of different dietary approaches on glycaemic control in patients with type 2 diabetes mellitus
Source: Eur J Epidemiol. 2018 Jan 4;33(2):157–70. doi: 10.1007/s10654-017-0352-x (PMC5871653; doi:10.1007/s10654-017-0352-x)
Supplement: Supplementary file 1 — Supplementary material 1 (PDF 384 kb) [file 10654_2017_352_MOESM1_ESM.pdf]

**Content**	**Page**
Supplementary Appendix S1: Search Strategy	2
Supplementary Figure S1: Flow diagram	3
Supplementary Table S1: Full-text articles excluded	4-5
Supplementary Figure S2: Risk of bias	6
Supplementary Table S2-3: Study characteristics	7-29
Supplementary Figure S3-6: Box plots	30-32
Supplementary Figure S7-8: Intervalplots	33-36
Supplementary Figure S9-10: Rankograms	37-38
Supplementary Figure S11-12: Loop-specific approach	39-40
Supplementary Table S4-5: Side-splitting approach	41-42
Supplementary Table S6-13: Subgroup and sensitivity analysis for network meta-analysis	43-52
Supplementary Figure S11: Meta-regression	53
Supplementary Figure S14-15: Comparison-adjusted funnel plot	54-55
Supplementary Figure S16: Bar graphs	56
Supplementary Appendix S2: GRADE assessment	57-58
Supplementary References of excluded studies	59-62

Supplementary Appendix S1:

PubMed:

(diabetes [MeSH Terms])

AND

(low-carbohydrate OR high-carbohydrate OR low-fat OR high-fat OR low-protein OR high-protein OR vegetarian OR vegan OR Mediterranean OR DASH OR dietary approaches to stop hypertension OR glycaemic index OR glycaemic load OR Palaeolithic OR low-calorie OR atkins)

AND

(glycemic OR glycaemia OR glycaemic OR glycemia OR HbA1c OR A1c OR glycated OR glycosylated OR glucose)

AND (random*)

Cochrane CENTRAL

(diabetes)

AND

(low-carbohydrate OR high-carbohydrate OR low-fat OR high-fat OR low-protein OR high-protein OR vegetarian OR vegan OR Mediterranean OR DASH OR dietary approaches to stop hypertension OR glycaemic index OR glycaemic load OR Palaeolithic OR low-calorie OR atkins)

AND

(glycemic OR glycaemia OR glycaemic OR glycemia OR HbA1c OR A1c OR glycated OR glycosylated OR glucose)

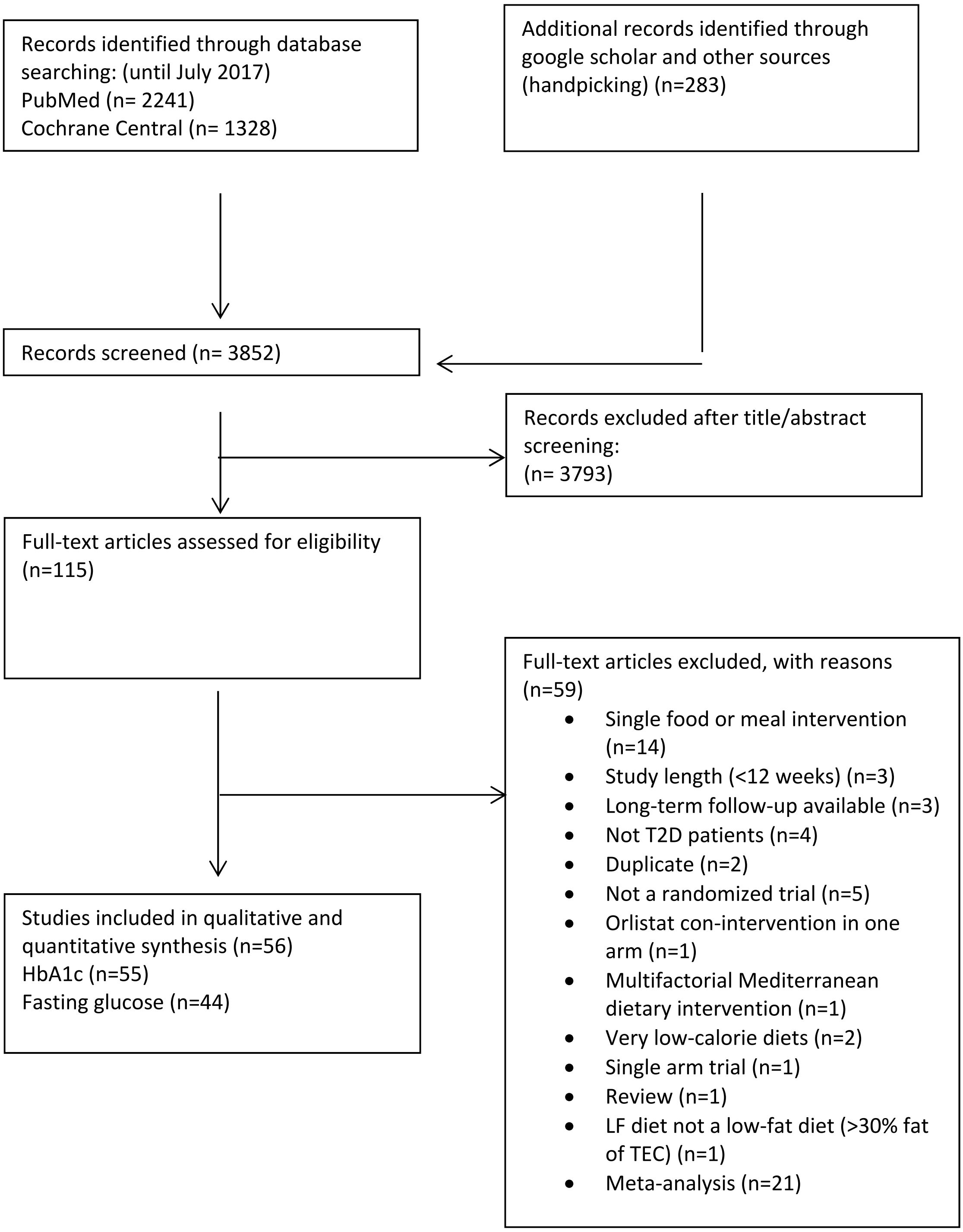

Supplementary Figure S1: Flow diagram of study selection

Reference	Reason for exclusion
[1]	Single food or meal intervention
[2]	Single food or meal intervention
[3]	Single food or meal intervention
[4]	Study length <12 weeks
[5]	Long-term follow-up available
[6]	Not T2D patients
[7]	Same study already included
[8]	Single food or meal intervention
[9]	Not a randomized trial
[10]	Study length <12 weeks
[11]	Study length <12 weeks
[12]	Not a randomized trial
[13]	Not a randomized trial
[14]	Single food or meal intervention
[15]	Not T2D patients
[16]	Single food or meal intervention
[17]	Orlistat co-intervention in one group
[18]	Single food or meal intervention
[19]	Single food or meal intervention
[20]	Not a randomized trial
[21]	Not a randomized trial
[22]	Not T2D patients
[23]	Single food or meal intervention
[24]	Single food or meal intervention
[25]	Long-term follow-up available
[26]	Multifactorial Mediterranean dietary intervention
[27]	Single food or meal intervention
[28]	Single food or meal intervention
[29]	Very low calorie diets
[30]	Very low calorie diets
[31]	Long-term follow-up available
[32]	Single-arm
[33]	Single food or meal intervention
[34]	Single food or meal intervention
[35]	Not T2D patients
[36]	Same study already included
[37]	LF diet not a low-fat diet (>30% fat of TEC)
[38]	Meta-analysis
[39]	Meta-analysis
[40]	Meta-analysis
[41]	Meta-analysis
[42]	Meta-analysis
[43]	Meta-analysis
[44]	Review
[45]	Meta-analysis
[46]	Meta-analysis
[47]	Meta-analysis
[48]	Meta-analysis
[49]	Meta-analysis
[50]	Meta-analysis
[51]	Meta-analysis
[52]	Meta-analysis

[53]	Meta-analysis
[54]	Meta-analysis
[55]	Meta-analysis
[56]	Meta-analysis
[57]	Meta-analysis
[58]	Meta-analysis
[59]	Meta-analysis

Supplementary Table S1: Full-text articles excluded, with reasons (n=59)

	Random sequence generation (selection bias)	Allocation concealment (selection bias)	Blinding of participants and personnel (performance bias)	Incomplete outcome data (attrition bias)	Selective reporting (reporting bias)
Andrews 2011	+	+	−	+	+
Barnard 2009	+	?	−	?	+
Brand 1994	?	?	−	+	+
Brehm 2009	?	?	−	+	+
Brinkworth 2004	?	?	−	−	?
Brunerova 2007	?	?	−	?	+
Ceriello 2014	+	?	−	+	−
Coppell 2010	+	?	−	+	+
Daly 2005	+	?	−	?	−
Davis 2009	+	?	−	+	?
de Bont 1981	?	?	−	?	+
Dyson 2007	+	?	−	?	?
Elhayany 2010	?	?	−	?	+
Esposito 2009	+	+	?	+	+
Fabricatore 2011	?	?	−	−	+
Guldbrand 2012	+	+	−	+	+
Heilbronn 1999	?	?	−	+	+
Hockaday 1978	?	?	−	+	?
Huang 2010	+	?	−	+	+
Iqbal 2009	+	?	−	−	+
Itsiopoulos 2011	?	?	−	?	+
Jenkins 2008	+	?	−	+	+
Jenkins 2012	+	?	−	+	+
Jenkins 2014	+	?	−	+	+
Jönsson 2009	+	?	−	+	+
Kahleova 2011	?	?	−	+	+
Kaplan 1987	?	?	−	+	?
Krebs 2012	+	+	?	?	+
Larsen 2011	+	+	?	+	+
Lasa 2014	+	+	?	+	+
Lee 2016	+	?	−	+	+
Li 2016	?	?	−	+	+
Liu 2016	?	?	+	?	+
Luger 2013	?	?	−	+	+
Ma 2008	+	?	−	?	+
McLaughlin 2007	?	?	−	+	?
Milne 1994	?	?	−	?	?
Mishra 2013	+	?	−	−	−
Nicholson 1999	?	?	−	+	+
Parker 2002	?	?	−	+	+
Pedersen 2014	+	+	−	−	+
Pritchard 1999	?	+	−	+	?
Rock 2014	+	?	−	+	+
Saslow 2014	+	+	−	+	+
Sato 2016	+	?	−	+	?
Shai 2008	+	+	?	+	+
Shige 2000	?	?	−	+	+
Stern 2004	+	+	−	?	+
Tay 2015	+	+	−	+	+
Uusitupa 1993	?	?	−	?	+
Walker 1995	?	?	−	?	+
Watson 2016	+	?	−	?	+
Westman 2008	+	?	−	+	+
Wolever 2008	+	+	−	+	+
Wycherley 2010	?	?	−	+	+
Yamada 2014	+	?	−	+	+

Supplementary Figure S2: Risk of bias of individual trials

Reference	Country	Study Design	Comparison Diets	Sample size	Mean age	Mean baseline BMI	Mean baseline HbA1c	Female (%)	Duration, months	Diet 1	Diet 2	Diet 3	Energy restricted	Drop out
Andrews 2016	United Kingdom	RCT, parallel	Low-fat vs. Control	347	Low Fat: 60.1 Control: 59.5	Low Fat: 31.5 Control: 32.3	Low Fat: 6.64 Control: 6.72	Low Fat: 36% Control: 37%	12	Low fat: Based on Diabetes UK dietary guidelines: choose foods in the lower ranges of energy density, fat content, and glycaemic index	Control: Standard dietary and exercise advice after randomization and at the end of the study, with reviews by a study doctor and nurse at baseline and at 6 and 12 months	NA	Low fat: yes Control: unrestricted	Low fat: 1% Control: 7%
Barnard 2009	USA	RCT, parallel	Vegetarian vs. Low-fat	99	Vegetarian: 56.7 Low-fat: 54.6	Vegetarian: 33.9 Low fat: 35.9	Vegetarian: 8.0 Low fat: 7.9	Vegetarian: 55% Low fat: 66%	18	Vegetarian: 75% carbohydrate, 15% protein, 10% fat	Low-fat: American Diabetes Association guidelines: 60-70% carbohydrate, 15-20% protein, <7% saturated fat and MUFA, <200 mg cholesterol	NA	Vegetarian: unrestricted Low-fat: diet with BMI >25: 500-1000 kcal/d	Vegetarian: 33%; Low fat: 24%
Brand 1994	Australia	RCT, crossover	Low glycaemic index/load vs. Low-fat	16	62	25	7.7	38%	3	Low glycaemic index/load: 6% of carbohydrate from legumes, 10% from porridge oats, 21% from pasta	Low-fat: 12% of carbohydrate from ready-to-eat breakfast cereals, 8% from potatoes, 8% from bananas	NA	No	No
Brehm 2009	USA	RCT, parallel	Moderate-carbohydrate vs. Low-fat	124	56.5	35.9	7.3	64%	12	Moderate carbohydrate: 45% carbohydrate, 15% protein, 40% fat, 20% MUFA	Low-fat: 60% carbohydrate, 15% protein, 25% fat	NA	200 to 300 kcal/d energy deficit	Moderate carbohydrate: 31%; Low-fat: 16%
Brinkworth 2004	Australia	RCT, parallel	High protein vs. Low-fat	64	High protein: 60.9 Low-fat: 62.7	High protein: 33.6 Low-fat: 33.3	High protein: 6.5 Low-fat: 6.2	NA	15	High protein: 40% carbohydrate, 30% protein, 30% fat	Low-fat: 55% carbohydrate, 15% protein, 30% fat	NA	No	High protein: 39% Low-fat: 42%
Brunerova 2007	Czech Republic	RCT, parallel	Moderate-carbohydrate vs. Low-fat	27	Moderate carbohydrate: 54.7 Low fat: 51.2	Moderate carbohydrate: 33.4 Low fat: 34.7	Moderate carbohydrate: 7.3 Low fat: 6.9	NA	3	Moderate carbohydrate: 45% carbohydrates, 10% protein, and 45% fat	Low fat: 60% carbohydrates, 10% protein, 30% fat	NA	600 kcal/d energy deficit	Moderate carbohydrate: NA Low fat: NA

Ceriello 2014	Italy	RCT, parallel	Mediterranean vs. Low-fat	24	NA	Mediterranean: 29.8<br>Low-fat: 29.2	Mediterranean: 8.1<br>Low-fat: 8.0	29%	3	Mediterranean: Enriched with 50mL MUFAs, Mediterranean diet recommendations	Low-fat: Recommendations to reduce all types of fat	NA	No	0%
Coppell 2010	New Zealand	RCT, parallel	Low-fat vs. Control	93	Low-fat: 56.6<br>Control: 58.4	Low-fat: 35.1<br>Control: 34.2	Low-fat: 8.9<br>Control: 8.6	59%	6	Low-fat: Advice on PA, 45-60% carbohydrate, 20g/1000 kcal of fibre, half of fibre should be soluble, 10-20% protein, < 30% fat: <10% saturated fat (or <8% if LDL was high, PUFA < 10%)	Control: Advice on PA, no change in diet	NA	Low-fat: At least 5% weight loss for overweight or obese	Low-fat: 11%;<br>Control: 8%
Daly 2005	United Kingdom	RCT, parallel	Low-carbohydrate vs. Low-fat	102	Low-carbohydrate: 58.2<br>Low-fat: 59.1	Low-carbohydrate: 35.4<br>Low-fat: 36.7	Low-carbohydrate: 9.0<br>Low-fat: 9.11	52%	3	Low-carbohydrate: Up to 70g carbohydrates/d including 1/2 pint milk and 1 fruit	Low-fat: Standard healthy eating advice, focused on reducing fat intake and reducing portion sizes	NA	No	23%
Davis 2009	USA	RCT, parallel	Low-carbohydrate vs. Low-fat	105	Low-carbohydrate: 54<br>Low-fat: 53	Low-carbohydrate: 35<br>Low-fat: 37	Low-carbohydrate: 7.5<br>Low-fat: 7.4	78%	12	Low-carbohydrate: 2 weeks carbohydrate restriction of 20-25g/d. 5g increment/week	Low-fat: 25% fat	NA	No	19%
de Bont 1981	United Kingdom	RCT, parallel	Moderate-carbohydrate vs. Low-fat	148	Moderate-carbohydrate: 54<br>Low-fat: 56	NA	Moderate-carbohydrate: 10.1<br>Low-fat: 10	100%	6	Moderate-carbohydrate: Goal of ≤40% carbohydrate	Low-fat: goal of 30% fat	NA	No	7%
Dyson 2007	United Kingdom	RCT, parallel	Low-carbohydrate vs. Low-fat	12	56	Low-carbohydrate: 36.5<br>Low fat: 33.3	Low-carbohydrate: 7.2<br>Low fat: 7.5	70%	3	Low-carbohydrate: 40 g/day and were given specific advice to take at least 200 ml of milk daily and include 4–5 portions of fruit and vegetables daily, with an emphasis on low-carbohydrate vegetables such as salads and green leafy vegetables	Low fat: Dietary guidelines of Diabetes UK	NA	Low-carbohydrate: unrestricted<br>Low fat: 500 kcal/d energy deficit	NA

Elhayany 2010	Israel	RCT, parallel	Mediterranean vs. Low-fat	259	Mediterranean: 56<br>Low-fat: 56	Mediterranean: 31<br>Low-fat: 31.8	Mediterranean: 8.3<br>Low-fat: 8.3	48%	12	Mediterranean 1:<br>50% carbohydrate, 30g fibre, 20% protein, 30% fat: of which 10% MUFA, 12% PUFA and 7% SFA<br>Mediterranean II:<br>35% carbohydrate, 30g fibre, 20% protein, 45% fat: of which 23% MUFA, 15% PUFA and 7% SFA	Low-fat:<br>50% carbohydrates, 15g fibre, 30% fat - of which 10% MUFA, 12% PUFA and 7% SFA - 20% protein	NA	No	25%
Esposito 2009	Italy	RCT, parallel	Mediterranean vs. Low-fat	215	Mediterranean: 52.4<br>Low-fat: 51.9	Mediterranean: 29.7<br>Low-fat: 29.5	Mediterranean: 7.75<br>Low-fat: 7.71	51%	48	Mediterranean:<br>Rich in vegetables, whole grains, low in red meat - replaced with poultry and fish - 30-50g olive oil; ≥50% complex carbohydrates, ≤30% fat	Low-fat<br>Rich in whole grains and restricted additional fats, sweets, high-fat snacks; ≤30% fat: ≤10% saturated fat	NA	1500 kcal/d women, 1800 kcal/d men	9%
Fabricatore 2011	USA	RCT, parallel	Low glycaemic index/load vs. Low-fat	79	Low-fat: 52.5<br>Low-GL: 52.8	Low-fat: 35.8<br>Low-GL: 36.7	Low-fat: 7.0<br>Low-GL: 6.6	80%	9	Low-fat:<br>≤30% fat	Low-GL:<br>≤3 and ≤1 servings/d of moderate-GL and high-GL foods, respectively	NA	No	37%
Guldbrand 2012	Sweden	RCT, parallel	Low-carbohydrate vs. Low-fat	61	Low-carbohydrate: 61.2<br>Low-fat: 62.7	Low-carbohydrate: 31.6<br>Low-fat: 33.8	Low-carbohydrate: 7.5<br>Low-fat: 7.2	56%	24	Low-carbohydrate:<br>20% carbohydrate, 30% protein, 50% fat	Low-fat:<br>55-60% carbohydrate, 10-15% protein, 30% fat: ≤10% saturated fat	NA	1600 kcal/d women, 1800 kcal/d men	0%
Heilbronn 1999	Australia	RCT, parallel	Moderate-carbohydrate vs. Low-fat	25	Moderate carbohydrate: 58.7<br>Low-fat: 57.5	Moderate carbohydrate: 33.6<br>Low-fat: 32.6	Moderate carbohydrate: 7.75<br>Low-fat: 8.51	80%	3	Moderate carbohydrate:<br>49% carbohydrates, 33% fat, 18% protein	Low-fat:<br>73% carbohydrates, 10% fat, 17% Protein	NA	Energy restricted	0%
Hockaday 1978	United Kingdom	RCT, parallel	Moderate-carbohydrate vs. Low-fat	93	Moderate-carbohydrate: 53<br>Low-fat: 50	NA	NA	44%	12	Moderate-carbohydrate:<br>40% carbohydrate, 20% protein, 40% fat: 28% SFA and MUFA, 12% PUFA	Low-fat:<br>54% carbohydrate, 20% protein, 26% fat: 10% SFA and MUFA, 16% PUFA	NA	1500 kcal/d	NA

Huang 2010	Taiwan	RCT, parallel	Low-fat vs. Control	154	Low-fat: 56 Control: 57	Low-fat: 25.7 Control: 27.0	Low-fat: 8.0 Control: 8.4	56%	12	Low-fat: An individualized diet plan was created to maintain intake of protein, fat, and carbohydrate energy to 15–20, 25–30, and 50–60%	Control: Control group received the routine care practiced at their primary care, which may have also included a summary of basic dietary principles by Nurses	NA	NA	Low-fat: 24% Control: 27%
Iqbal 2009	USA	RCT, parallel	Low-carbohydrate vs. Low-fat	144	59.4	37.6	Low-carbohydrate: 7.9 Low-fat: 7.6	10%	24	Low-carbohydrate: Goal of 30g/d carbohydrates; more whole grains and unsaturated FAs encouraged	Low-fat: <7% saturated fats, <300 mg cholesterol, encouraged to increase fruits and vegetable intake	NA	Low-fat: 500 kcal/d energy deficit	53% (weight was retrieved from medical records for 75% of drop outs)
Itsiopoulos 2011	Australia	RCT, crossover	Mediterranean vs. Low-fat	31	59	30.7	7.1	41%	3	Mediterranean: 44% carbohydrate, 12% protein, 40% fat: >50% from MUFA - , 4% alcohol from red wine, 47g/d fibre; 70% of foods provided by the study	Low-fat: No alteration of diet 27% fat	NA	No	13%
Jenkins 2008	Canada	RCT, parallel	Low glycaemic index/load vs. Low-fat	210	Low glycaemic index/load: 60 Low-fat: 61	Low glycaemic index/load: 30.6 Low-fat: 31.2	7.1	39%	6	Low glycaemic index/load: 42-43% carbohydrate; from low-GI foods list in prescribed servings of 15g	Low-fat: 42-43% carbohydrate; from high-cereal fibre foods list in prescribed servings of 15g	NA	No	26%
Jenkins 2012	Canada	RCT, parallel	Low glycaemic index/load vs. Low-fat	121	Low glycaemic index/load: 58 Low fat: 61	Low glycaemic index/load: 31.4 Low fat: 29.9	Low glycaemic index/load: 7.4 Low fat: 7.2	Low glycaemic index/load: 47% Low fat: 52%	3	Low glycaemic index/load: legume consumption was 1 cup per day approximately 190 g per day, or 2 servings per day) of cooked beans, chickpeas or lentils	Low fat: Whole wheat and whole grain carbohydrate foods: whole wheat breakfast cereals, breads, brown rice	NA	NA	Low glycaemic index/load: 6% Low fat: 5%

Jenkins 2014	Canada	RCT, parallel	Low glycaemic index/load vs. Low-fat	141	Low glycaemic index/load: 59<br>Low fat: 59	Low glycaemic index/load: 30<br>Low fat: 31	Low glycaemic index/load: 7.4<br>Low fat: 7.2	Low glycaemic index/load: 46%<br>Low fat: 45%	3	Low glycaemic index/load: included: 4.5 slices of canola oil–enriched whole-wheat bread (500 kcal/d) provided as a supplement; low-GI foods, including legumes, barley, pasta, parboiled rice, and temperate-climate fruit, as outlined in previous studies	Low fat: 7.5 slices of whole-wheat bread without canola oil per day (500 kcal); participants were instructed to avoid white-flour products and replace them with whole-wheat breakfast cereals, study breads, brown rice, and so forth	NA	NA	Low glycaemic index/load: 21%<br>Low fat: 10%
Jönsson 2009	Sweden	RCT, crossover	Palaeolithic vs. Low-fat	13	64	30	6.6	23%	6	Palaeolithic: Based on lean meat, fish, fruit, vegetables, eggs and nuts, excluding dairy's, cereal grains, beans, refined fats, sugar, candy, soft drinks, beer and added salt	Low-fat: Increased vegetable, fibre, whole-grain cereal products and fruits; decreased total fat, saturated fat and salt	NA	No	0%
Kahleova 2011	Czech Republic	RCT, parallel	Vegetarian vs. Low-fat	74	Vegetarian: 54.6<br>Low-fat: 57.7	Vegetarian: 35.1<br>Low-fat: 35	Vegetarian: 7.6<br>Low-fat: 7.7	53%	6	Vegetarian: 60% carbohydrate, 15% protein, 25% fat; animal products limited to 1 portion of low-fat yogurt/day	Low-fat: 50% carbohydrate, 20% protein, <30% fat: ≤7% SFA, <200mg cholesterol	NA	500 kcal/d energy deficit	16%
Kaplan 1987	USA	RCT, parallel	Low-fat vs. Control	76	Low fat: 57<br>Control: 54	NA	Low fat: 9.18<br>Control: 8.16	NA	18	Low Fat: 50% carbohydrate, 20% protein, 30% fat	Control: Standard dietary advice	NA	1200 kcal/d	NA
Krebs 2012	New Zealand	RCT, parallel	High-Protein vs. Low-fat	419	58	36.6	8.1	60%	12	High-Protein: 40% carbohydrate, 30% protein, 30% fat	Low-fat: 55% carbohydrate, 15% protein, 30% fat	NA	500 kcal/d energy deficit	30%
Larsen 2011	Australia	RCT, parallel	High-Protein vs. Low-fat	99	High-Protein: 59.6<br>Low-fat: 58.8	27-49	High-Protein: 7.89<br>Low-fat: 7.78	52%	12	High-Protein: 40% carbohydrate, 30% protein, 30% fat	Low-fat: 55% carbohydrate, 15% protein, 30% fat	NA	1500 kcal /d diet, or 30% energy restriction for 3 months; followed by 9 months of energy balance	7%

Lasa<br>2014	Spain	RCT,<br>parallel	Mediterranean<br>vs.<br>Low-fat	191	Mediterranean:<br>67<br>Low-fat:<br>67.2	Mediterranean:<br>29.8<br>Low-fat:<br>29.8	NA	60%	12	Mediterranean 1:<br>Mediterranean diet + 1L/week of virgin olive oil<br>Mediterranean 2:<br>Mediterranean diet + 30g/d mixed nuts	Low fat:<br><30% fat	NA	No	no
Lee<br>2016	Korea	RCT,<br>parallel	Vegetarian<br>vs.<br>Low-fat	93	Vegetarian:<br>57.5<br>Low-fat:<br>58.3	Vegetarian:<br>23.9<br>Low-fat:<br>23.1	Vegetarian:<br>7.7<br>Low fat:<br>7.4	37%	3	Vegetarian:<br>Whole grains, vegetables, fruit, and legumes	Low fat<br>(50–60% carbohydrate, 15–20% protein<br>(if renal function is normal), <25% fat)	NA	Low fat:<br>iso-caloric	Vegan:<br>13%<br>Low fat:<br>11%
Li<br>2016	China	RCT,<br>parallel	Low-fat<br>vs.<br>Control	139	Low fat:<br>59<br>Control:<br>59	Low fat:<br>27.19<br>Control:<br>25.17	Low fat:<br>8.10<br>Control:<br>8.05	48%	12	Low-fat:<br>Carbohydrate, 22% fat, 18% from protein	Control:<br>No dietary intervention	NA	Low-fat:<br>2275 kcal d for men And 1890 kcal/d for women	Low fat:<br>4%<br>Control:<br>2%
Liu<br>2016	China	RCT,<br>Parallel	Low-fat<br>vs.<br>Control	117	Low fat:<br>63.3<br>Control:<br>62	Low fat:<br>25.8<br>Control:<br>26.8	Low fat:<br>7.55<br>Control:<br>7.78	61%	12	Low-fat:<br>Food groups are assigned colours of the traffic light. RED light (stop and think): fat, processed meats, cake, and sugar-sweetened drinks.<br>YELLOW light (good for you, but watch portion size): grains, meat, poultry, fish and shellfish, nuts, eggs, and oil.<br>GREEN light (go, low in calorie-density and high in nutrients): vegetables, fruits, dairy, beans and legumes, and water. The TLD guide is provided to patients with type 2 diabetes to help them make healthier food choices	Control:<br>Routine care practiced at their community health service centres, which may have included a summary of basic dietary principles by clinicians	NA	NA	Low fat:<br>10%<br>Control:<br>8%

Luger 2013	Austria	RCT, parallel	High-protein vs. Low-fat	44	High-protein: 61 Low-fat: 63.7	High-protein: 33 Low-fat: 33.6	High-protein: 7.8 Low-fat: 7.6	54%	3	High-protein: 30 % protein 40 % carbohydrates: soy-based foods (e. g. tofu), milk products, fish and poultry	Low-fat: 15 % protein and 55 % carbohydrates	NA	NA	NA
Ma 2008	USA	RCT, parallel	Low glycaemic index/load vs. Low-fat	40	53.5	35.8	Low glycaemic index/load: 8.74 Low-fat: 8.10	53%	12	Low glycaemic index/load: 55% carbohydrate, which were ranked according to GI	Low-fat: 55% carbohydrate, counting - all treated the same	NA	No	0%
McLaughlin 2007	USA	RCT, parallel	Moderate-carbohydrate vs. Low-fat	29	Moderate-carbohydrate: 57 Low-fat: 56	Moderate-carbohydrate: 31.4 Low-fat: 31	NA	41%	4	Moderate-carbohydrate: 40% carbohydrate, 15% protein, 45% fat	Low-fat: 60% carbohydrate, 15% protein, 25% fat	NA	750 kcal/d energy deficit	0%
Milne 1994	New Zealand	RCT, parallel	Moderate-carbohydrate vs. Low-fat vs. Control	70	Moderate-carbohydrate: 59 Low-fat: 60 Control: 5 8	Moderate-carbohydrate: 29 Low-fat: 30 Control: 29	10	45%	18	Moderate carbohydrate: 45% carbohydrate, 19% protein, 36% fat; SFA:PUFA:MUFA = 1	Low-fat: 55% carbohydrate, ≥30g/d fibre, increase soluble fibre, 15% protein, 30% fat: ≤10 SFA	Control	500 kcal/d for BMI >25 energy deficit	9%
Mishra 2013	USA	RCT, parallel	Vegetarian vs. Low-fat	291	Vegetarian: 44.3 Low-fat: 46.1	Vegetarian: 34.7 Low-Fat: 35.3	Vegetarian: 7.54 Low-Fat: 7.05	83%	4	Vegetarian Avoiding all animal products, <3g of fat from added oils/serving, favour low-GI foods	Low-fat	NA	No	28%
Nicholson 1999	USA	RCT, parallel	Vegetarian vs. Low-fat	13	Vegetarian: 51 Low-fat: 60	NA	Vegetarian: 8.3 Low-fat: 8.0	46%	3	Vegetarian: Whole grains, vegetables, legumes and fruits; 10-15% protein, <10% fat, rest carbohydrates	Low-fat: Fish and poultry rather than red meat; 55-60% carbohydrate, <30% fat, 200mg/d cholesterol	NA	No	15%

Parker 2002	Australia	RCT, parallel	High-protein vs. Low-fat	54	High-protein men: 63.4<br>High-protein women: 58.7<br>Low-fat men: 64.2<br>Low-fat women: 60.9	High-protein men: 35.4<br>High-protein women: 34.5<br>Low-fat men: 33.4<br>Low-fat women: 33.2	High-protein: 6.42<br>Low-fat: 6.30	65%	3	High-protein:<br>42% carbohydrate, 28% protein, 28% fat: 8% SFA, 12% MUFA, 5% PUFA	Low-fat:<br>55% carbohydrate, 16% protein, 26% fat: 8% SFA, 11% MUFA, 5% PUFA)	NA	8 weeks 1600 kcal/d, 4 weeks energy balance	0%
Pedersen 2014	Australia	RCT, parallel	High-protein vs. Low-fat	76	High-protein: 59.4 Low-fat: 62.4	High-protein: 36.7<br>Low-fat: 35.4	High-protein: 7.5<br>Low-fat: 7.1	22%	12	High-protein:<br>40% carbohydrate, 30% protein, 30% fat	Low-fat<br>(50% carbohydrate, 20% protein, 30% fat)	NA	1430 kcal/d	41%
Pritchard 1999	Australia	RCT, parallel	Low-fat vs. Control	17	NA	NA	NA	NA	12	Low fat:<br>>50% carbohydrates, <30% fat	Control: no intervention	NA	NA	Low fat: 28%<br>Control: 0%
Rock 2014	USA	RCT, parallel	Moderate-carbohydrate vs. Low-fat vs. Control	227	56	Moderate-carbohydrate: 36.2<br>Low-fat: 36<br>Control: 36.3	Moderate-carbohydrate: 7.3<br>Low-fat: 7.5<br>Control: 7.4	51%	12	Moderate-carbohydrate<br>45% carbohydrate, 25% protein, 30% fat	Low-fat:<br>60% carbohydrate, 20% protein, 20% fat	Control: Counselling sessions with advice for 500-1000 kcal/d deficit and Dietary Guidelines for Americans: 55% carbohydrates, 15% protein, 30% fat	Moderate-carbohydrate and Low-Fat: 1200-2000 kcal/d	10%

Saslow 2014	USA	RCT, parallel	Low-carbohydrate vs. Moderate carbohydrate	34	Low-carbohydrate: 64.8 Moderate carbohydrate: 55.1	Low-carbohydrate: 26.7 Moderate carbohydrate: 26.5	Low-carbohydrate: 6.6 Moderate carbohydrate: 6.9	Low-carbohydrate: 56% Moderate carbohydrate: 89%	3	Low-carbohydrate: Reduce carbohydrate intake over 7–10 days to between 20–50 grams of carbohydrates a day, not including fibre: referred to as net grams of carbohydrates; with the goal of achieving nutritional ketosis	Moderate carbohydrate: Low fat, calorie-restricted, carbohydrate counting diet consistent with guidelines from American Diabetes Association, 45% calories from carbohydrates	NA	Low-carbohydrate: unrestricted Low fat: -500 kcal/d energy deficit	0%
Sato 2016	Japan	RCT, parallel	Moderate-carbohydrate vs. Low-fat	62	Moderate carbohydrate: 60.5 Low fat: 58.4	Moderate carbohydrate: 26.7 Low fat: 26.5	Moderate carbohydrate: 8 Low fat: 8.3	24%	6	Moderate carbohydrate: 130g/d carbohydrates	Low fat: 50-60% carbohydrates	NA	unrestricted	Moderate carbohydrate: 9% Low fat: 3%
Shai 2008	Israel	RCT, parallel	Mediterranean vs. Low-fat vs. Low-carbohydrate	36	NA	NA	NA	14%	24	Mediterranean: 35% fat adding olive oil and nuts, 2 portions fish/week	Low-fat: 30% fat, 10% SFA, 300mg cholesterol	Low-carbohydrate (based on Atkins diet; aim 20g/d carbohydrates for 2 months and gradual increase to weight loss maintenance level)	Low-fat and Mediterranean (increasing restriction, unspecified)	15%
Shige 2000	Australia	RCT, parallel	Moderate-carbohydrate vs. Low-fat	24	Moderate carbohydrate: 58.1 Low fat: 57.5	Moderate carbohydrate: 33.1 Low fat: 32.6	Moderate carbohydrate: 7.9 Low fat: 8.5	79%	3	Moderate carbohydrate: 50% carbohydrate, 18% protein, and 32% fat	Low fat: 73% carbohydrates, 17% protein, 9% fat	NA	30% energy restriction	0%
Stern 2004	USA	RCT, parallel	Low-carbohydrate vs. Low-fat	54	Low-carbohydrate: 53 Low-fat: 54	Low-carbohydrate: 42.9 Low-fat: 42.9	Low-carbohydrate: 7.4 Low-fat: 7.3	17%	12	Low-carbohydrate: <30g/d carbohydrates	Low-fat: <30% fat	NA	Low-fat diet: 500 kcal/d energy deficit	34%
Tay 2015	Australia	RCT, parallel	Low-carbohydrate vs. Low-fat	115	58	34.6	7.3	43%	12	Low-carbohydrate: 14% carbohydrate (<50g), 28% protein, 58% fat: <10% SFA	Low-fat: 53% carbohydrate, 17% protein, 30% fat: <10% SFA	NA	Diets were matched for energy with 500-1000 kcal/d deficit	33%

Uusitupa 1993	Sweden	RCT, parallel	Low-fat vs. Control	86	Low-fat men: 50.7 Low-fat women: 53.7 Control men: 54.0 Control women: 54.4	Low-fat: 33.2 Control: 32.7	Low-fat: 8.4 Control: 9.0	43%	12	Low-fat: ≤30% fat: <10% SFA, cholesterol <250-300 mg/d, increase in unrefined carbohydrates, regular eating patterns	Control: Usual diet	NA	Low-Fat: individually planned energy restriction	0% (4 drop-outs excluded from analysis)
Walker 1995	Australia	RCT, crossover	Moderate-carbohydrate vs. Low-fat	24	58	29.2	Moderate-carbohydrate: 6.4 Low fat: 6.8	63%	3	Moderate carbohydrate: 40% carbohydrate, 20% protein, 40% fat	Low-fat: 59% carbohydrate, 20% protein, 21% fat	NA	Iso-caloric	NA
Watson 2016	Australia	RCT, parallel	High-protein vs. Low-fat	61	High-protein: 54 Low-fat: 55	High-protein: 34.3 Low fat: 34.4	High-protein: 8 Low fat: 8.1	High-protein: 47% Low fat: 45%	3	High-protein: 38% carbohydrate, 30% protein, 29% fat	Low-fat: 53% carbohydrate, 21% protein, 23% fat	NA	Iso-caloric	High-protein: 28% Low fat: 28%
Westman 2008	USA	RCT, parallel	Low-carbohydrate vs. Low glycaemic index/load	84	Low-carbohydrate: 51.8 Low glycaemic index/load: 51.8	Low-carbohydrate: 37.7 Low glycaemic index/load :38.5	Low-carbohydrate: 8.8 Low glycaemic index/load: 8.3	79%	6	Low-carbohydrate: <20g carbohydrates, unlimited amounts of animal foods and eggs, limited cheese and vegetables	Low glycaemic index/load: 55% carbohydrate, low-GI foods	NA	Low glycaemic index/load: 500 kcal/d energy deficit	42%
Wolever 2008	Canada	RCT, parallel	Low glycaemic index/load vs. Low-fat vs. Moderate-carbohydrate	162	Low glycaemic index/load: 60.6 Moderate-carbohydrate: 58.6 Low-fat: 60.4	Low glycaemic index/load: 31.6 Moderate-carbohydrate: 31.1 Low-fat: 30.1	Low glycaemic index/load: 6.2 moderate-carbohydrate: 6.1 Low-fat: 6.2	54%	12	Low glycaemic index/load: GI of 55; 52% carbohydrate, 21% protein, 27% fat	Moderate-carbohydrate: GI of 59; 39% carbohydrate, 21% protein, 40% fat	Low-fat: GI of 63; 47% carbohydrate, 22% protein, 31% fat	Energy deficit 500 kcal/d if subject wished to lose weight	20%

Wycherley 2010	Australia	RCT, parallel	High-protein vs. Low-fat	83	56	35.4	High-protein: 6.8-8.0 Low-fat: 7.3-7.6	NA	4	High-protein: 43% carbohydrate, 33% protein, 22% fat	Low-fat: 53% carbohydrate, 19% protein, 26% fat	NA	Women 1400 kcal/d; Men 1700 kcal/d	29%
Yamada 2014	Japan	RCT, parallel	Moderate-carbohydrate vs. Low-fat	24	Moderate-carbohydrate: 63.3 Low-fat: 63.2	Moderate-carbohydrate: 24.5 Low-fat: 27	Moderate-carbohydrate: 7.6 Low fat: 7.7	50%	6	Moderate-carbohydrate <130 g/day; lower limit of carbohydrate intake to 70 g/d	Low-fat: Carbohydrates50-60%, protein 20% and fat 25%	NA	NA	0%

Supplementary Table S2: General study characteristics of the included trials investigating the effects of different dietary approaches on glycaemic control

d: Day; FA: fatty acids; GI: glycaemic index; GL: glycaemic load; kcal: kilocalorie; LDL-C: low-density lipoprotein cholesterol; MUFA: monounsaturated fat; NA: not applicable; PA: physical activity; PUFA: polyunsaturated fat; RCT: randomized controlled trial; SFA: saturated fat

Reference	**Comparison Diets**	Presence of comorbidities	Hypoglycaemic drugs (%)	Antihypertensive medication (%)	Lipid lowering medication (%)	Risk of bias	Body weight
Andrews 2016	Control	NA	35	59	64	L	89
	Low-fat	NA	40	68	65	L	94
Barnard 2009	Vegetarian	18 (eye involvement), 12 (renal involvement), 37 (neuropathy)	69 (metformin), 22 (insulin), 51 (sulfonylurea), 33 (thiazolidinedione), 2 (other)	63	55	H	93
	Low-fat	20 (eye involvement), 8 (renal involvement), 48 (neuropathy)	78 (metformin), 10 (insulin), 58 (sulfonylurea), 30 (thiazolidinedione), 4 (other)	76	54	H	96
Brand 1994	Low glycaemic index/load	NA	63 (oral hypoglycaemic agents)	NA	NA	H	76
	Low-fat	NA	64 (oral hypoglycaemic agents)	NA	NA	H	76
Brehm 2009	Moderate-carbohydrate	NA	NA	NA	NA	H	100
	Low-fat	NA	NA	NA	NA	H	98
Brinkworth 2004	High-Protein	NA	NA	NA	NA	H	92
	Low-fat	NA	NA	NA	NA	H	89
Brunerova 2007	Moderate-carbohydrate	NA	NA	NA	NA	H	90
	Low-fat	NA	NA	NA	NA	H	98

Ceriello 2014	Mediterranean	NA	NA	NA	NA	H	NA
	Low-fat	NA	NA	NA	NA	H	NA
Coppell 2010	Control		71 (oral hypoglycaemic agents) 0 (insulin) 29 (oral hypoglycaemic agents and insulin)	63	48	L	96
	Low-fat		69 (oral hypoglycaemic agents) 1 (insulin) 29 (oral hypoglycaemic agents and insulin)	53	31	L	95
	Low-carbohydrate	NA	40 (oral hypoglycaemic agent), 20 (insulin), 40 (oral hypoglycaemic agent + insulin)	NA	NA	H	55
Daly 2005	Low-fat	NA	41 (oral hypoglycaemic agent), 20 (insulin), 40 (oral hypoglycaemic agent + insulin)	NA	NA	H	58
	Low-carbohydrate	NA	78 (metformin), 44 (sulfonylurea), 35 (insulin)	NA	62	H	91
Davis 2009	Low-fat	NA	86 (metformin), 52 (sulfonylurea), 24 (insulin)	NA	56	H	98
de Bont 1981	Moderate-carbohydrate	NA	65 (oral hypoglycaemic drugs), 2 (insulin)	NA	NA	H	72
	Low-fat	NA	62 (oral hypoglycaemic	NA	NA	H	72

			drugs), 1 (insulin)				
Dyson 2007	Low-carbohydrate	NA	NA	NA	NA	H	92
	Low-fat	NA	NA	NA	NA	H	96
Elhayany 2010	Mediterranean	NA	NA	NA	NA	H	78
	Low-fat	NA	NA	NA	NA	H	80
Esposito 2009	Mediterranean	NA	NA	24	15	L	82
	Low-fat	NA	NA	23	16	L	83
Fabricatore 2011	Low glycaemic index/load	NA	NA	NA	NA	H	96
	Low-fat	NA	NA	NA	NA	H	92
Guldbrand 2012	Low-carbohydrate	NA	50 (oral glucose-lowering medication), 33 (oral glucose-lowering medication+ insulin)	NA	NA	L	89
	Low-fat	NA	42 (oral glucose-lowering medication), 35 (oral glucose-lowering medication+ insulin)	NA	NA	L	96
Heilbronn 1999	Moderate-carbohydrate	NA	49 (oral hypoglycaemic	NA	NA	H	NA

			agents)				
	Low-fat	NA	50 (oral hypoglycaemic agents)	NA	NA	H	NA
Hockaday 1978	Moderate-carbohydrate	NA	NA	NA	NA	H	73
	Low-fat	NA	NA	NA	NA	H	78
Huang 2010	Control	NA	95 (sulfonylurea), 83 (biguanide), 23 (thiazolidinedione)	81	22	L	NA
	Low-fat	NA	96 (sulfonylurea), 80 (biguanide), 13 (thiazolidinedione)	61	40	L	NA
Iqbal 2009	Low-carbohydrate	27 (coronary artery disease), 7 (heart failure)	85 (oral medications), 30 (insulin)	63	46	H	116
	Low-fat	26 (coronary artery disease), 15 (heart failure)	85 (oral medications), 23 (insulin)	69	62	H	115
Itsiopoulos 2011	Mediterranean	NA	56 (oral hypoglycaemic agents), 11 (insulin)	48	26	H	NA
	Low-fat	NA	57 (oral hypoglycaemic agents), 11 (insulin)	48	26	H	NA
Jenkins 2008	Low glycaemic index/ load	NA	100 (anti-hyperglycaemic medications)	66	67	L	85
	Low-fat	NA	98 (anti-hyperglycaemic medications)	65	60	L	86

Jenkins 2012	Low glycaemic index/load	NA	100 (anti-hyperglycaemic medications)	72	73	L	83
	Low-fat	NA	100 (anti-hyperglycaemic medications)	61	67	L	86
Jenkins 2014	Low glycaemic index/load	NA	100 (anti-hyperglycaemic medications)	56	71	L	83
	Low-fat	NA	100 (anti-hyperglycaemic medications)	61	72	L	82
Jönsson 2009	Palaeolithic	NA	69 (metformin), 23 (sulfonylurea), 23 (thiazolidinedione)	37	62	L	81
	Low-fat	NA	70 (metformin), 23 (sulfonylurea), 23 (thiazolidinedione)	37	62	L	84
Kahleova 2011	Vegetarian	NA	76 (metformin), 35 (sulfonylurea), 14 (thiazolidinedione), 8 (other)	68	59	H	95
	Low-fat	NA	79 (metformin), 54 (sulfonylurea), 19 (thiazolidinedione), 22 (other)	59	43	H	98
Kaplan 1987	Low-fat	NA	76 (oral hypoglycaemic agents), 50 (insulin)	NA	NA	H	NA
	Control	NA	77 (oral hypoglycaemic	NA	NA	H	NA

			agents), 50 (insulin)				
Krebs 2012	High-protein	NA	56 (oral agents), 25 (insulin + oral agents)	77	62	L	100
	Low-fat	NA	57 (oral agents), 29 (insulin + oral agents)	75	69	L	96
Larsen 2011	High-protein	NA	74 (oral agents), 15 (insulin)	NA	NA	L	92
	Low-fat	NA	73 (oral agents), 19 (insulin)	NA	NA	L	93
Lasa 2014	Mediterranean	NA	NA	NA	NA	L	NA
	Low-fat	NA	NA	NA	NA	L	NA
Lee 2016	Vegetarian	13 (history of eye involvement)	74 (metformin), 37 (sulfonylurea), 17 (insulin), 30 (other)	39	50	L	NA
	Low-fat	13 (history of eye involvement)	77 (metformin), 45 (sulfonylurea), 17 (insulin), 40 (other)	47	55	L	NA
Li 2016	Control	NA	53 (oral diabetic drugs), 20 (insulin), 18 (combined treatment)	NA	NA	H	71
	Low-fat	NA	57 (oral diabetic drugs), 18 (insulin), 16 (combined treatment)	NA	NA	H	73

	Control	NA	NA	NA	NA	H	67
Liu 2016	Low-fat	NA	NA	NA	NA	H	65
Luger 2013	High-protein	NA	NA	NA	L	91	NA
	Low-fat	NA	NA	NA	L	91	NA
Ma 2008	Low glycaemic index/load	NA	73 (metformin), 38 (glyburide), 25 (hypoglycaemic agents + insulin)	NA	NA	H	94
	Low-fat	NA	73 (metformin), 38 (glyburide), 25 (hypoglycaemic agents + insulin)	NA	NA	H	101
	Moderate-carbohydrate	NA	NA	NA	NA	H	89
McLaughlin 2007	Low-fat	NA	NA	NA	NA	H	83
Milne 1994	Moderate-carbohydrate	50 (hypoglycaemic drugs)	NA	NA	H	82	NA
	Low-fat	57 (hypoglycaemic drugs)	NA	NA	H	80	NA

	Control	52 (hypoglycaemic drugs)	NA	NA	H	80	NA
Mishra 2013	Vegetarian	NA	NA	NA	NA	H	94
	Low-fat	NA	NA	NA	NA	H	96
Nicholson 1999	Vegetarian	14 (coronary artery bypass)	86 (hypoglycaemic agents), 29 (insulin)	86	NA	H	90
	Low-fat	25 (coronary artery bypass)	100 (hypoglycaemic agents), 0 (insulin)	100	NA	H	93
Parker 2002	High-Protein	NA	48 (hypoglycaemic agents), 7 (insulin)	NA	NA	H	92
	Low-fat	NA	49 (hypoglycaemic agents), 7 (insulin)	NA	NA	H	87
Pedersen 2014	High-Protein	NA	91 (oral agents or insulin)	100	96	L	98
	Low-fat	NA	92 (oral agents or insulin)	100	96	L	98

Pritchard 1999	Control	NA	NA	NA	NA	H	NA
	Low-fat	NA	NA	NA	NA	H	NA
Rock 2014	Moderate-carbohydrate	79 (hypertension), 70 (high cholesterol), 5 (Coronary artery disease)	90 (oral hypoglycaemic agents), 13 (insulin)	84	68	L	97
	Low-fat	62 (hypertension), 62 (high cholesterol), 7 (Coronary artery disease)	84 (oral hypoglycaemic agents), 26 (insulin)	70	66	L	98
	Control	75 (hypertension), 74 (high cholesterol), 1 (Coronary artery disease)	82 (oral hypoglycaemic agents), 16 (insulin)	79	75	L	102
Saslow 2014	Low-carbohydrate	63 (Hypertension), 81 (dyslipidaemia)	31 (metformin), 44 (metformin + another diabetes agent)	NA	NA	L	95
	Moderate-carbohydrate	78 (Hypertension), 56 (dyslipidaemia)	44 (metformin), 28 (metformin + another diabetes agent)	NA	NA	L	97
Sato 2016	Moderate-carbohydrate	NA	63 (metformin), 37 (sulfonylureas), 23 (insulin)	37	66	H	68
	Low-fat	NA	66 (metformin), 44 (sulfonylureas), 40 (insulin)	41	69	H	73
Shai 2008	Mediterranean	NA	NA	NA	NA	L	NA

	Low-fat	NA	NA	NA	NA	L	NA
	Low-carbohydrate	NA	NA	NA	NA	L	NA
Shige 2000	Moderate-carbohydrate	NA	NA	NA	NA	H	84
	Low-fat	NA	NA	NA	NA	H	83
Stern 2004	Low-carbohydrate	NA	NA	NA	NA	L	NA
	Low-fat	NA	NA	NA	NA	L	NA
Tay 2015	Low-carbohydrate	NA	79 (metformin), 34 (sulfonylurea), 5 (thiazolidinedione), 10 (insulin)	71	60	L	88
	Low-fat	NA	72 (metformin), 28 (sulfonylurea), 5 (thiazolidinedione), 11 (insulin)	61	63	L	90
Uusitupa 1993	Control	7 (myocardial infarction), 17 (angina pectoris), 9 (cardiac failure), 46 (hypertension)	NA	NA	NA	H	NA
	Low-fat	8 (myocardial infarction), 18 (angina pectoris), 10 (cardiac failure), 48 (hypertension)	NA	NA	NA	H	NA

Walker 1995	Moderate-carbohydrate	NA	NA	NA	NA	H	79
	Low-fat	NA	NA	NA	NA	H	79
	High-Protein	NA	58 (metformin), 16 (sulfonylureas), 19 (insulin)	61	52	H	88
Watson 2016	Low-fat	NA	64 (metformin), 18 (sulfonylureas), 21 (insulin)	43	64	H	94
Westman 2008	Low glycaemic index/load	NA	76 (hypoglycaemic medications)	NA	NA	L	97
	Low-carbohydrate	NA	95 (hypoglycaemic medications)	NA	NA	L	98
Wolever 2008	Moderate-carbohydrate	NA	0	48	43	L	84
	Low-fat	NA	0	48	43	L	84
	Low glycaemic index/load	NA	0	48	43	L	84
Wycherley 2010	High-Protein	NA	NA	56	61	49	H
	Low-fat	NA	NA	56	61	49	H
Yamada 2014	Moderate-carbohydrate	17 (retinopathy), 100 (nephropathy)	42 (metformin), 42 (sulfonylureas), 25 (insulin)	NA	NA	L	64

	Low-fat	17 (retinopathy), 100 (nephropathy)	8 (metformin), 67 (sulfonylureas), 33 (insulin)	NA	NA	L	67

Supplementary Table S3: Study characteristics including presence of comorbidities, medication status, average risk of bias, and body weight of the included trials investigating the effects of different dietary approaches on glycaemic control

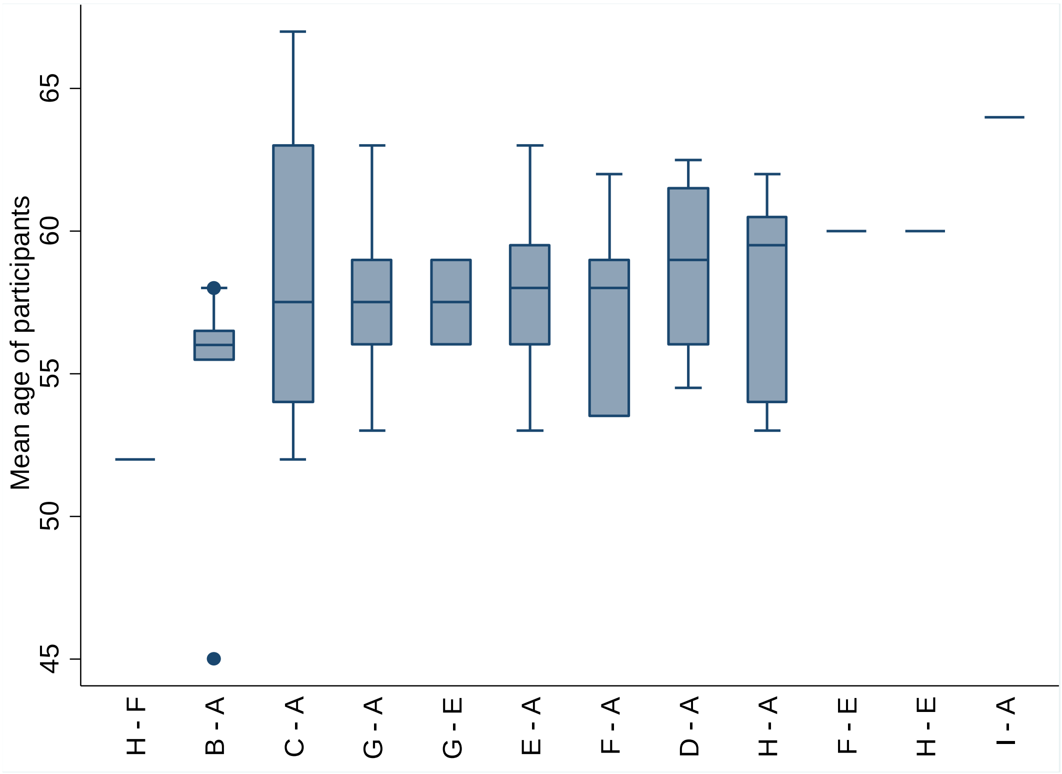

Supplementary Figure S3: Box plots showing the distribution of the mean age (years) of the trials across the available direct comparisons.

[A=Low-fat, B=Vegetarian, C=Mediterranean, D=High-Protein, E=Moderate-carbohydrate, F=Low-carbohydrate, G=control, H=Low GI/GL, I=Palaeolithic].

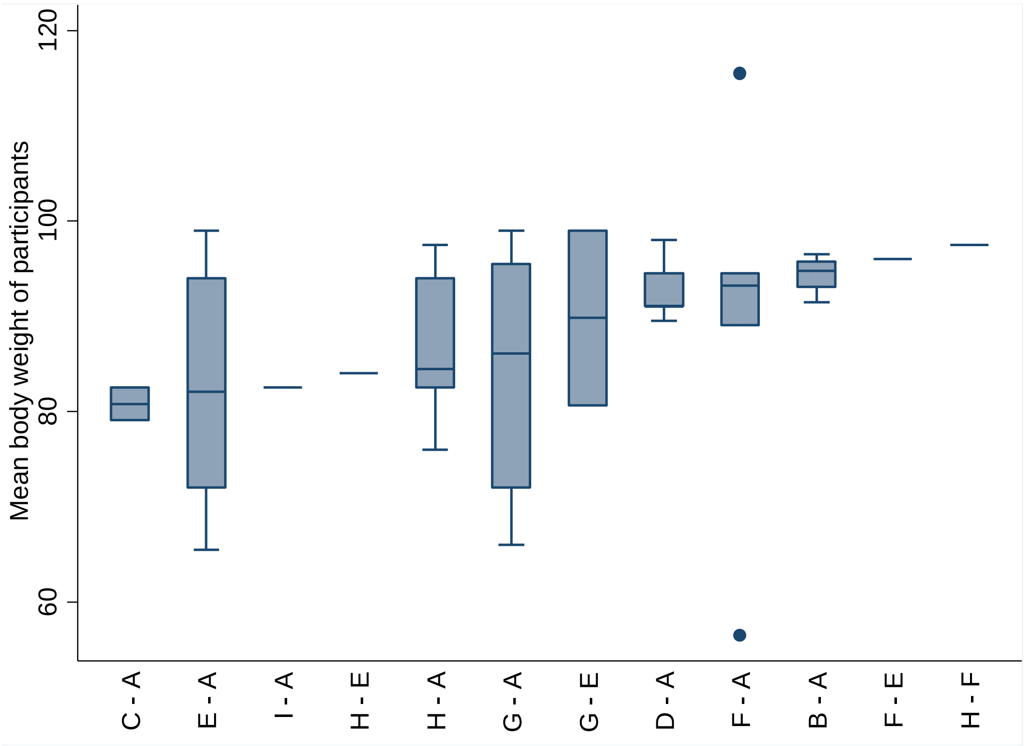

Supplementary Figure S4: Box plots showing the distribution of the mean body weight (kg) of the trials across the available direct comparisons.

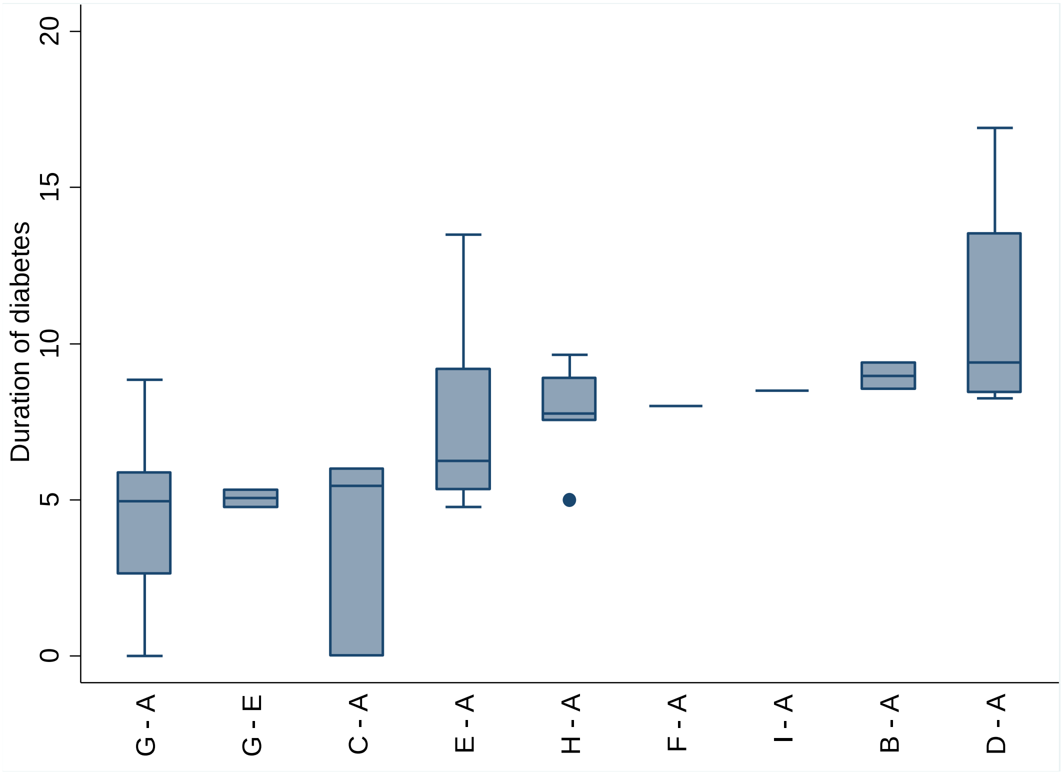

Supplementary Figure S5: Box plots showing the distribution of the mean duration of diabetes (years) in the trials across the available direct comparisons.

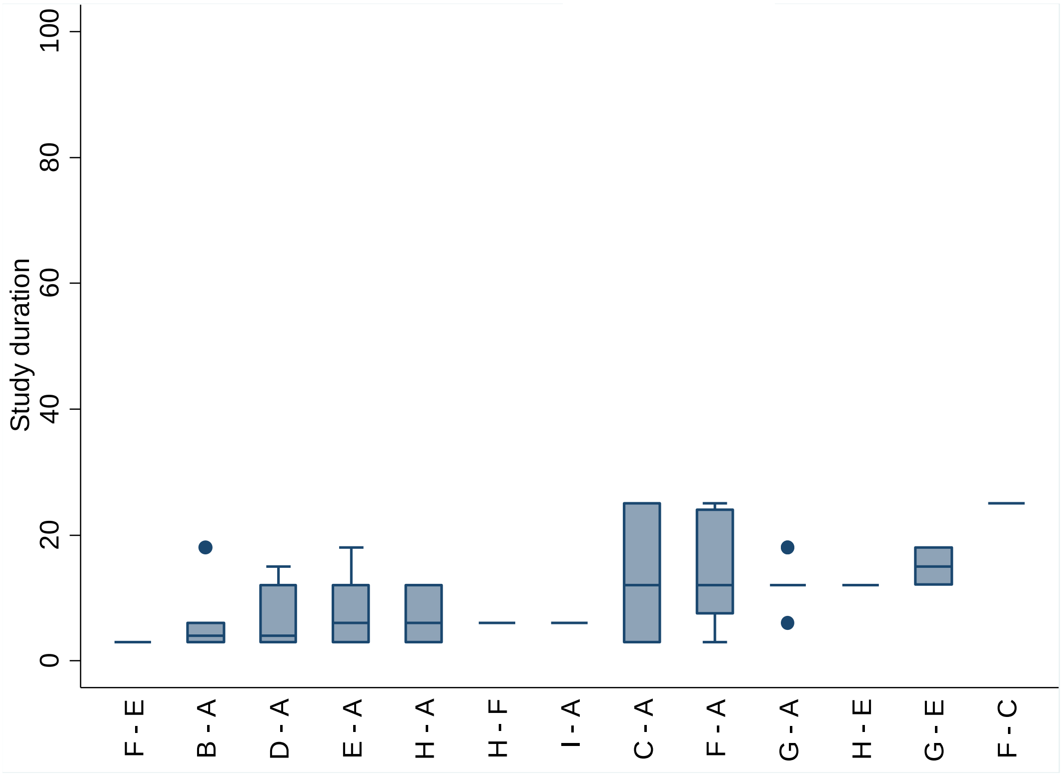

Supplementary Figure S6: Box plots showing the distribution of the trial duration (months) across the available direct comparisons.

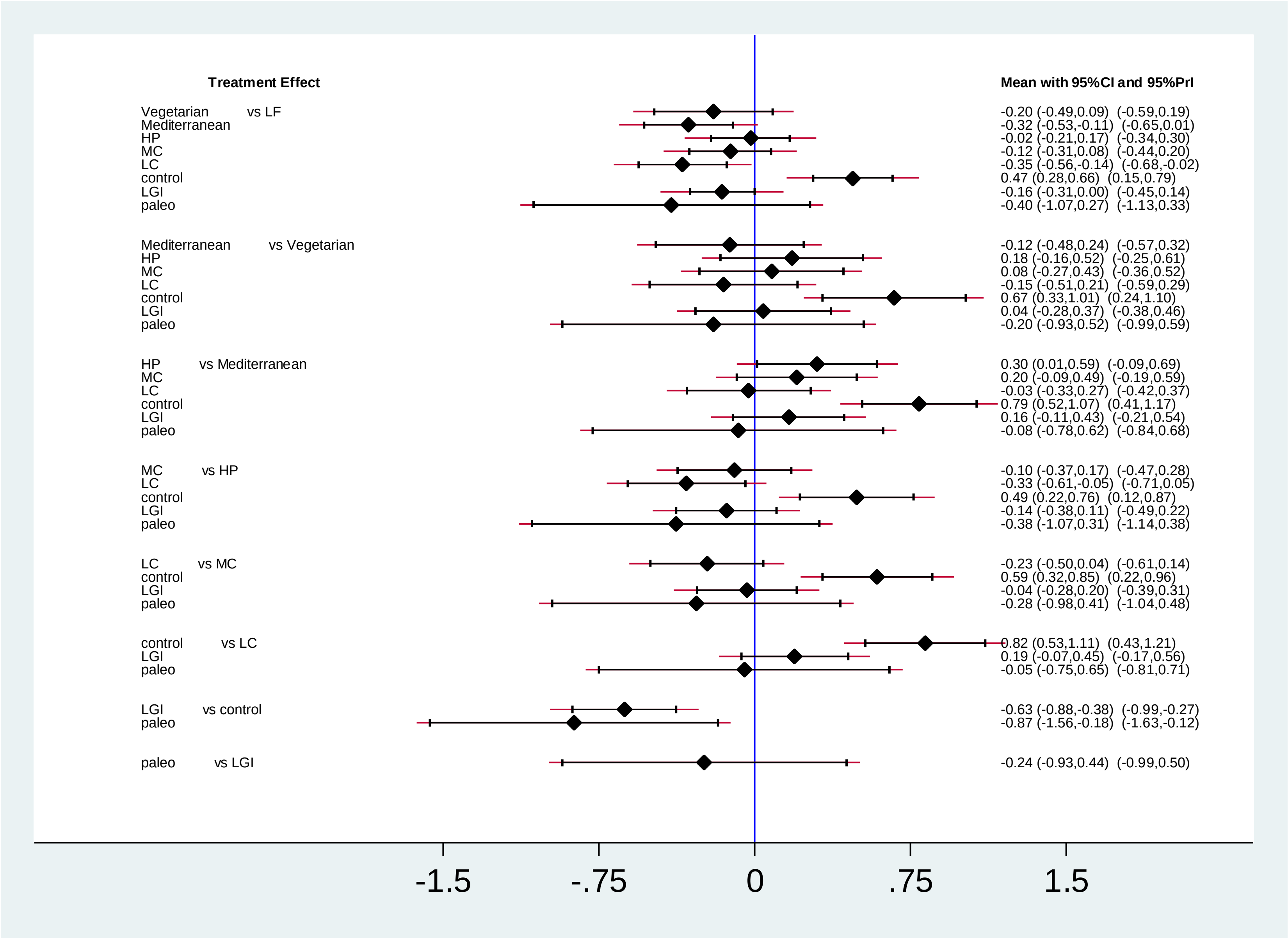
Treatment Effect
Mean with 95%CI and 95%PrI
Vegetarian vs LF
Mediterranean
HP
MC
LC
control
LGI
paleo
-0.20 (-0.49,0.09) (-0.59,0.19)
-0.32 (-0.53,-0.11) (-0.65,0.01)
-0.02 (-0.21,0.17) (-0.34,0.30)
-0.12 (-0.31,0.08) (-0.44,0.20)
-0.35 (-0.56,-0.14) (-0.68,-0.02)
0.47 (0.28,0.66) (0.15,0.79)
-0.16 (-0.31,0.00) (-0.45,0.14)
-0.40 (-1.07,0.27) (-1.13,0.33)
Mediterranean vs Vegetarian
HP
MC
LC
control
LGI
paleo
-0.12 (-0.48,0.24) (-0.57,0.32)
0.18 (-0.16,0.52) (-0.25,0.61)
0.08 (-0.27,0.43) (-0.36,0.52)
-0.15 (-0.51,0.21) (-0.59,0.29)
0.67 (0.33,1.01) (0.24,1.10)
0.04 (-0.28,0.37) (-0.38,0.46)
-0.20 (-0.93,0.52) (-0.99,0.59)
HP vs Mediterranean
MC
LC
control
LGI
paleo
0.30 (0.01,0.59) (-0.09,0.69)
0.20 (-0.09,0.49) (-0.19,0.59)
-0.03 (-0.33,0.27) (-0.42,0.37)
0.79 (0.52,1.07) (0.41,1.17)
0.16 (-0.11,0.43) (-0.21,0.54)
-0.08 (-0.78,0.62) (-0.84,0.68)
MC vs HP
LC
control
LGI
paleo
-0.10 (-0.37,0.17) (-0.47,0.28)
-0.33 (-0.61,-0.05) (-0.71,0.05)
0.49 (0.22,0.76) (0.12,0.87)
-0.14 (-0.38,0.11) (-0.49,0.22)
-0.38 (-1.07,0.31) (-1.14,0.38)
LC vs MC
control
LGI
paleo
-0.23 (-0.50,0.04) (-0.61,0.14)
0.59 (0.32,0.85) (0.22,0.96)
-0.04 (-0.28,0.20) (-0.39,0.31)
-0.28 (-0.98,0.41) (-1.04,0.48)
control vs LC
LGI
paleo
0.82 (0.53,1.11) (0.43,1.21)
0.19 (-0.07,0.45) (-0.17,0.56)
-0.05 (-0.75,0.65) (-0.81,0.71)
LGI vs control
paleo
-0.63 (-0.88,-0.38) (-0.99,-0.27)
-0.87 (-1.56,-0.18) (-1.63,-0.12)
paleo vs LGI
-0.24 (-0.93,0.44) (-0.99,0.50)
-1.5
-.75
0
.75
1.5

Supplementary Figure S7: Mean differences (MD) for glycosylated haemoglobin (%) as estimated from network meta-analysis for every possible pair of interventions. Solid lines represent 95% Cis, and red lines 95% PrI.

HP=High-Protein, LC=Low-carbohydrate, LF=Low-fat, LGI=Low GI/GL, MC=Moderate-carbohydrate

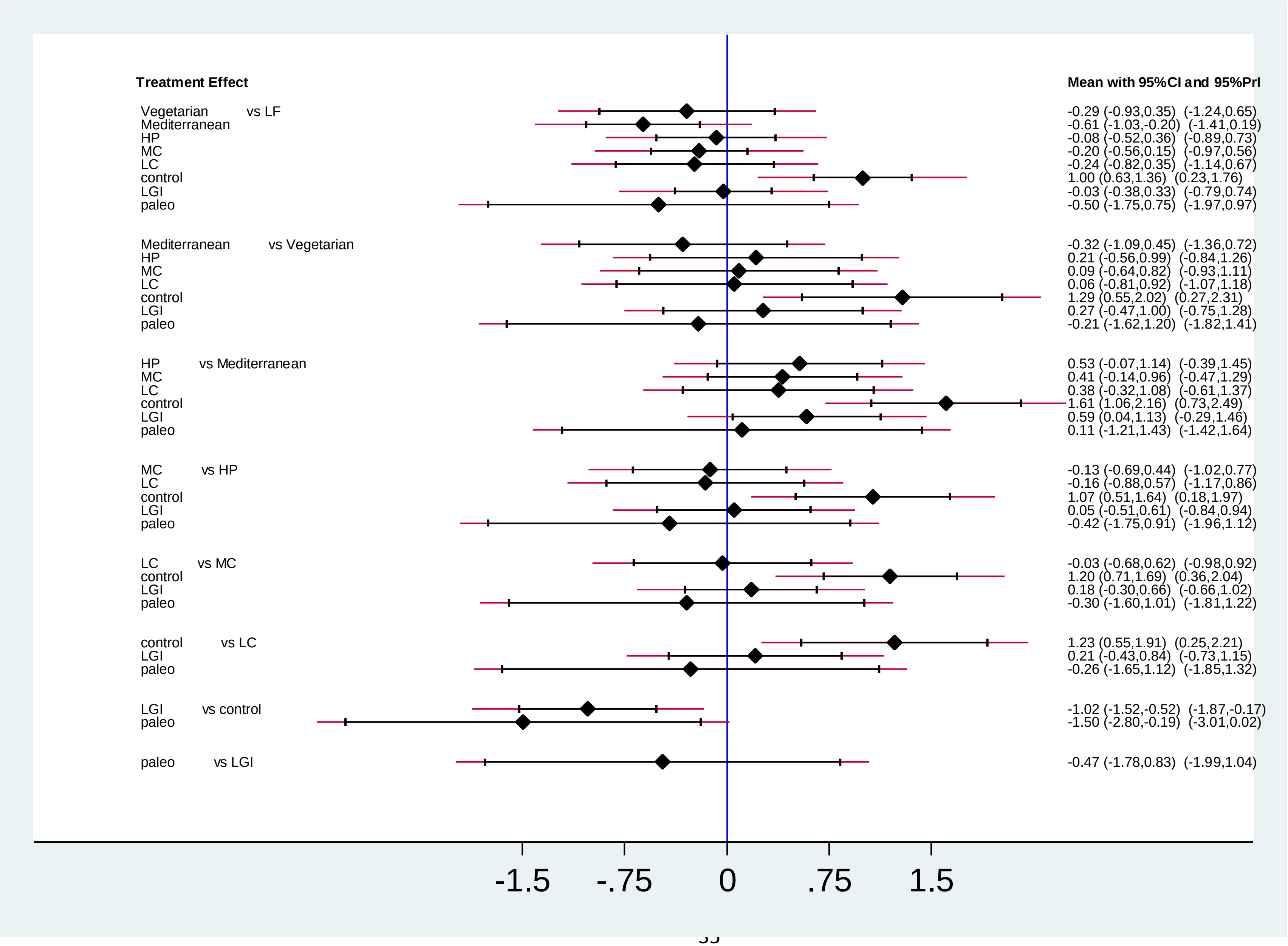

Treatment Effect
Mean with 95%CI and 95%PrI
Vegetarian vs LF
Mediterranean
HP
MC
LC
control
LGI
paleo
-0.29 (-0.93,0.35) (-1.24,0.65)
-0.61 (-1.03,-0.20) (-1.41,0.19)
-0.08 (-0.52,0.36) (-0.89,0.73)
-0.20 (-0.56,0.15) (-0.97,0.56)
-0.24 (-0.82,0.35) (-1.14,0.67)
1.00 (0.63,1.36) (0.23,1.76)
-0.03 (-0.38,0.33) (-0.79,0.74)
-0.50 (-1.75,0.75) (-1.97,0.97)
Mediterranean vs Vegetarian
HP
MC
LC
control
LGI
paleo
-0.32 (-1.09,0.45) (-1.36,0.72)
0.21 (-0.56,0.99) (-0.84,1.26)
0.09 (-0.64,0.82) (-0.93,1.11)
0.06 (-0.81,0.92) (-1.07,1.18)
1.29 (0.55,2.02) (0.27,2.31)
0.27 (-0.47,1.00) (-0.75,1.28)
-0.21 (-1.62,1.20) (-1.82,1.41)
HP vs Mediterranean
MC
LC
control
LGI
paleo
0.53 (-0.07,1.14) (-0.39,1.45)
0.41 (-0.14,0.96) (-0.47,1.29)
0.38 (-0.32,1.08) (-0.61,1.37)
1.61 (1.06,2.16) (0.73,2.49)
0.59 (0.04,1.13) (-0.29,1.46)
0.11 (-1.21,1.43) (-1.42,1.64)
MC vs HP
LC
control
LGI
paleo
-0.13 (-0.69,0.44) (-1.02,0.77)
-0.16 (-0.88,0.57) (-1.17,0.86)
1.07 (0.51,1.64) (0.18,1.97)
0.05 (-0.51,0.61) (-0.84,0.94)
-0.42 (-1.75,0.91) (-1.96,1.12)
LC vs MC
control
LGI
paleo
-0.03 (-0.68,0.62) (-0.98,0.92)
1.20 (0.71,1.69) (0.36,2.04)
0.18 (-0.30,0.66) (-0.66,1.02)
-0.30 (-1.60,1.01) (-1.81,1.22)
control vs LC
LGI
paleo
1.23 (0.55,1.91) (0.25,2.21)
0.21 (-0.43,0.84) (-0.73,1.15)
-0.26 (-1.65,1.12) (-1.85,1.32)
LGI vs control
paleo
-1.02 (-1.52,-0.52) (-1.87,-0.17)
-1.50 (-2.80,-0.19) (-3.01,0.02)
paleo vs LGI
-0.47 (-1.78,0.83) (-1.99,1.04)
-1.5
-.75
0
.75
1.5

Supplementary Figure S8: Mean differences (MD) for fasting glucose (mmol/l) as estimated from network meta-analysis for every possible pair of interventions. Solid lines represent 95% Cis, and red lines 95% PrI

HP=High-Protein, LC=Low-carbohydrate, LF=Low-fat, LGI=Low GI/GL, MC=Moderate-carbohydrate

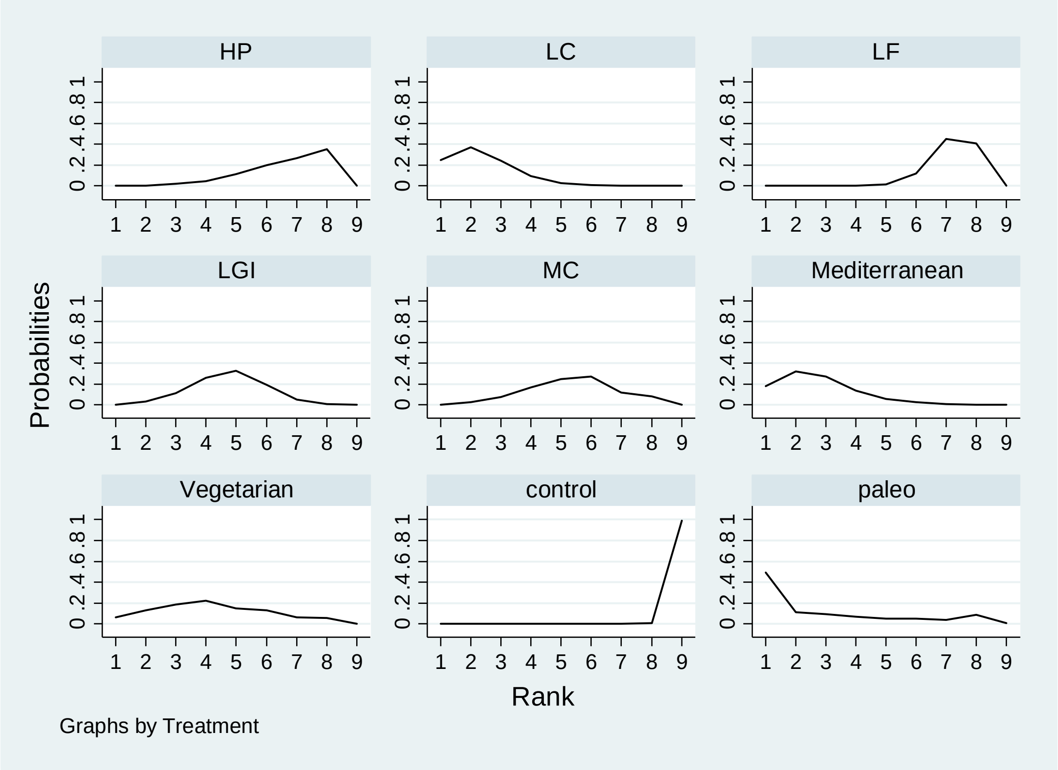

Supplementary Figure S9: Rankograms for HbA1c (%)

HP=High-Protein, LC=Low-carbohydrate, LF=Low-fat, LGI=Low GI/GL, MC=Moderate-carbohydrate

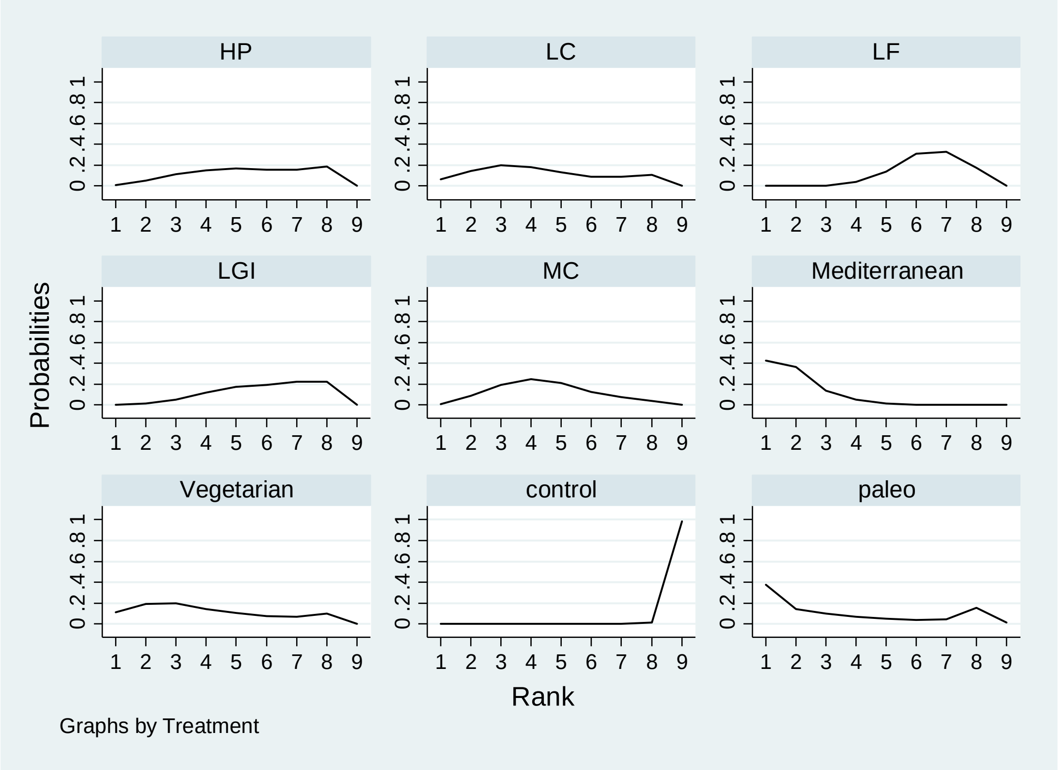

Supplementary Figure S10: Rankogram for fasting glucose (mmol/l)

HP=High-Protein, LC=Low-carbohydrate, LF=Low-fat, LGI=Low GI/GL, MC=Moderate-carbohydrate

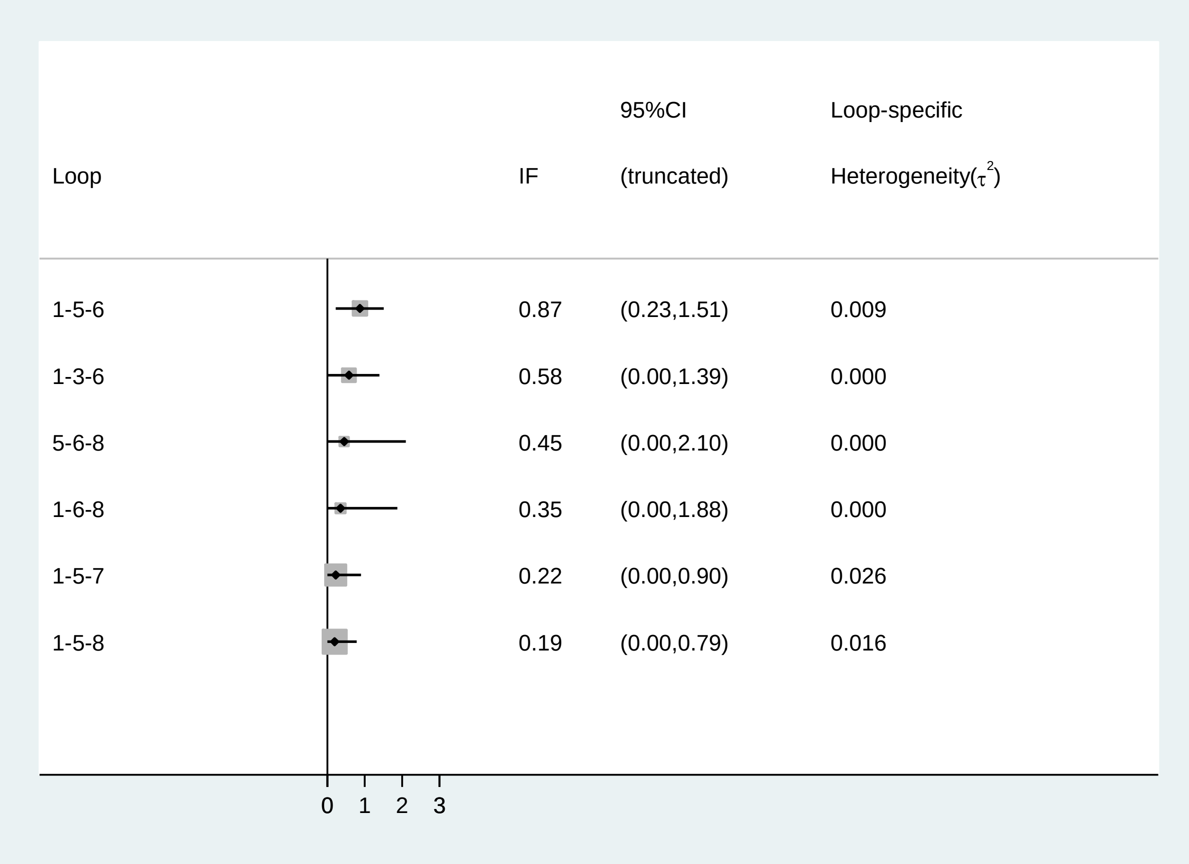

Supplementary Figure S11: Loop-specific approach for inconsistency for HbA1c

[1=Low-fat, 2=Vegetarian, 3=Mediterranean, 4=High-Protein, 5=Moderate-carbohydrate, 6=Low-carbohydrate, 7=control, 8=Low GI/GL, 9=Palaeolithic].

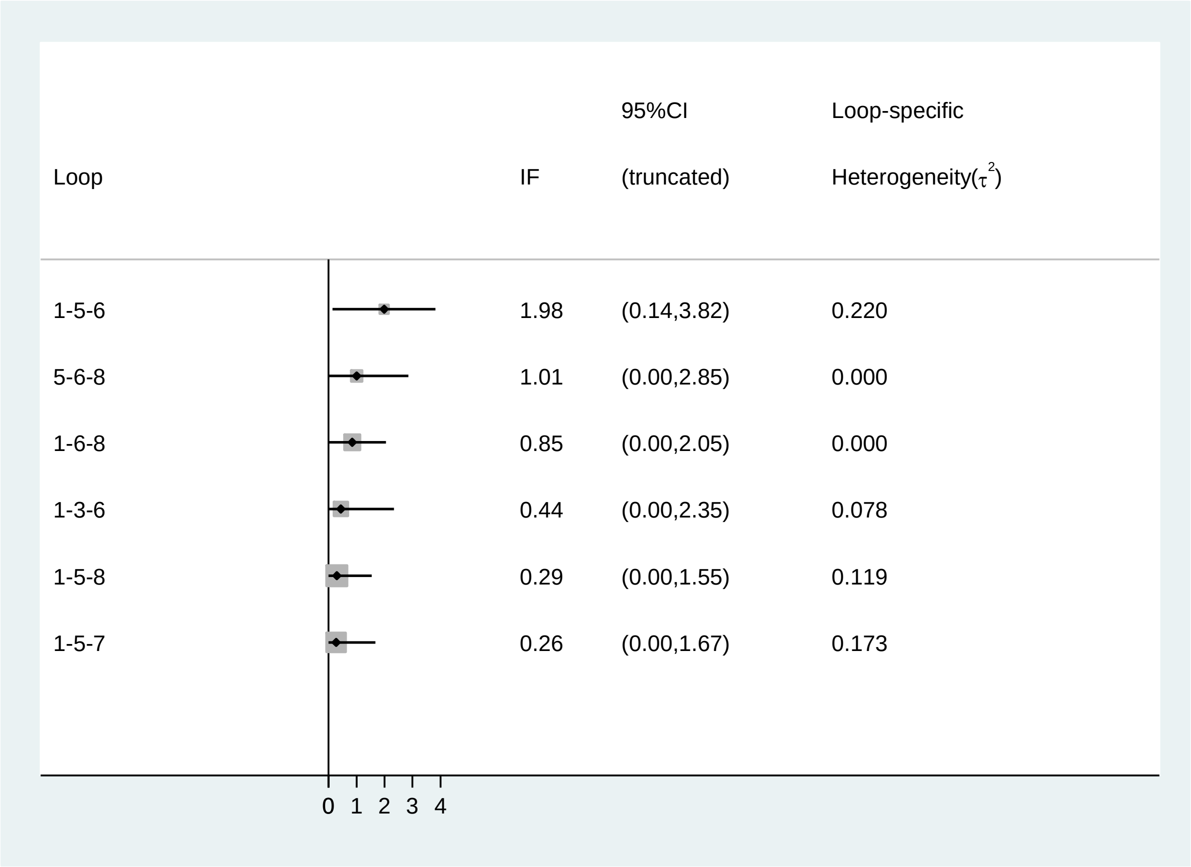

Supplementary Figure S12: Loop-specific approach for inconsistency for fasting glucose

[1=Low-fat, 2=Vegetarian, 3=Mediterranean, 4=High-Protein, 5=Moderate-carbohydrate, 6=Low-carbohydrate, 7=control, 8=Low GI/GL, 9=Palaeolithic].

Side	Direct		Indirect		Difference		
	Coef.	Std. Err.	Coef.	Std. Err.	Coef.	Std. Err.	P>|z|
1 3 *	-.3360849	.1084342	.2971034	.7375321	-.6331883	.7487571	0.398
1 5	-.1861283	.1113727	.1955237	.2409221	-.381652	.2664811	0.152
1 6	-.2134685	.1091256	-1.045446	.2621847	.8319771	.286296	0.004
1 7 *	.4205536	.0910826	1.401375	.4463529	-.9808214	.4558512	0.031
1 8	-.147641	.078519	-.4272755	.4007604	.2796344	.4073853	0.492
3 6	-.3959493	.4068855	.0277253	.1582971	-.4236747	.4366692	0.332
5 6	-.8999992	.2840959	-.0447772	.1471216	-.855222	.3199301	0.008
5 7	.9333773	.2648388	.4640484	.1514136	.4693288	.3138661	0.135
5 8	-.0595211	.2472088	-.0341048	.1452736	-.0254162	.2877383	0.930
6 8	.4099864	.7807053	.1854637	.1335653	.2245228	.7920484	0.777

Supplementary Table S4: Side-splitting approach for inconsistency for HbA1c

[1=Low-fat, 2=Vegetarian, 3=Mediterranean, 4=High-Protein, 5=Moderate-carbohydrate, 6=Low-carbohydrate, 7=control, 8=Low GI/GL, 9=Palaeolithic].

Side	Direct		Indirect		Difference		
	Coef.	Std. Err.	Coef.	Std. Err.	Coef.	Std. Err.	P>|z|
1 3 *	-.6122477	.2167232	-.59068	1.818422	-.0215677	1.838986	0.991
1 5	-.2799723	.1967195	.413155	.5633204	-.6931273	.5980085	0.246
1 6	.2385932	.3764301	-.9191882	.4522758	1.157781	.5824865	0.047
1 7 *	.95863	.1857425	2.089195	1.008676	-1.130565	1.02482	0.270
1 8	-.0285752	.1930546	.000853	.5894406	-.0294282	.6200235	0.962
3 6	.4784134	.8622421	.3553019	.390742	.1231116	.9384489	0.896
5 6	-1.459987	.725599	.3308306	.3641646	-1.790818	.8118549	0.027
5 7	1.549413	.569377	1.114961	.2781715	.4344523	.6340549	0.493
5 8	.1421199	.5448269	.1874224	.2815729	-.0453025	.6141079	0.941
6 8	.4999955	.6085481	.0940235	.3849339	.405972	.7200736	0.573

Supplementary Table S5: Side-splitting approach for inconsistency for fasting glucose

[1=Low-fat, 2=Vegetarian, 3=Mediterranean, 4=High-Protein, 5=Moderate-carbohydrate, 6=Low-carbohydrate, 7=control, 8=Low GI/GL, 9=Palaeolithic].

HbA1c (%)							
Low-Carb	0.09 (-0.26,0.45)	-0.14 (-0.78,0.51)	-0.23 (-0.72,0.25)	-0.12 (-0.52,0.27)	-0.25 (-0.64,0.14)	-0.22 (-0.49,0.05)	-0.75 (-1.10,-0.39)
	Mediterranean	-0.23 (-0.87,0.40)	-0.33 (-0.80,0.14)	-0.22 (-0.59,0.16)	-0.35 (-0.72,0.03)	-0.31 (-0.56,-0.06)	-0.84 (-1.15,-0.53)
		Vegetarian	-0.10 (-0.81,0.61)	0.01 (-0.64,0.67)	-0.12 (-0.77,0.53)	-0.08 (-0.67,0.51)	-0.61 (-1.23,0.02)
			Low-GI/GL	0.11 (-0.32,0.54)	-0.02 (-0.51,0.47)	0.02 (-0.38,0.42)	-0.51 (-0.96,-0.06)
				Moderate-Carb	-0.13 (-0.53,0.27)	-0.09 (-0.38,0.20)	-0.62 (-0.95,-0.29)
					High- Protein	0.04 (-0.24,0.32)	-0.49 (-0.85,-0.13)
						Low-Fat	-0.53 (-0.75,-0.31)
							Control

Supplementary Table S6: League table: Long-term studies: ≥12 months. The values above the diet classes correspond to the difference in mean (95% CI) in HbA1c (%) between the row and columns (eg, the mean difference in average HbA1c between Low-Carb and Low-Fat diet is -0.22%)

HbA1c (%)								
Low-Carb	-0.39 (-1.19,0.41)	-0.22 (-1.01,0.57)	-0.38 (-0.91,0.14)	-0.41 (-0.84,0.01)	-0.45 (-0.87,-0.04)	-0.54 (-1.02,-0.07)	-0.62 (-1.01,-0.23)	-0.82 (-1.49,-0.16)
	Mediterranean	0.17 (-0.81,1.15)	0.01 (-0.77,0.79)	-0.02 (-0.76,0.71)	-0.06 (-0.82,0.70)	-0.15 (-0.90,0.60)	-0.23 (-0.93,0.47)	-0.43 (-1.31,0.45)
		Palaeolithic	-0.16 (-0.93,0.61)	-0.19 (-0.91,0.53)	-0.23 (-0.98,0.52)	-0.32 (-1.06,0.42)	-0.40 (-1.09,0.29)	-0.60 (-1.47,0.27)
			Vegetarian	-0.03 (-0.44,0.38)	-0.07 (-0.53,0.39)	-0.16 (-0.60,0.29)	-0.24 (-0.59,0.11)	-0.44 (-1.08,0.20)
				Low-GI/GL	-0.04 (-0.40,0.32)	-0.13 (-0.47,0.22)	-0.21 (-0.43,0.01)	-0.41 (-0.99,0.17)
					Moderate-Carb	-0.09 (-0.50,0.32)	-0.17 (-0.47,0.14)	-0.37 (-0.99,0.25)
						High-Protein	-0.08 (-0.36,0.20)	-0.28 (-0.89,0.33)
							Low-Fat	-0.20 (-0.74,0.34)
								Control

Supplementary Table S7: League table: Short-term studies: <12 months. The values above the diet classes correspond to the difference in mean (95% CI) in HbA1c (%) between the row and columns (eg, the mean difference in average HbA1c between Low-Carb and Low-Fat diet is -0.62%)

HbA1c (%)						
Low-Carb	0.27 (-0.09,0.62)	0.01 (-0.31,0.34)	0.07 (-0.32,0.46)	-0.19 (-0.60,0.23)	-0.11 (-0.39,0.18)	-0.57 (-0.92,-0.21)
	Mediterranean	-0.25 (-0.51,0.00)	-0.19 (-0.53,0.14)	-0.45 (-0.82,-0.09)	-0.37 (-0.59,-0.16)	-0.83 (-1.12,-0.54)
		Low-GI/GL	0.06 (-0.23,0.35)	-0.20 (-0.53,0.13)	-0.12 (-0.27,0.03)	-0.58 (-0.83,-0.33)
			Moderate-Carb	-0.26 (-0.66,0.14)	-0.18 (-0.44,0.08)	-0.64 (-0.95,-0.33)
				High-Protein	0.08 (-0.22,0.38)	-0.38 (-0.74,-0.02)
					Low-Fat	-0.46 (-0.67,-0.25)
						Control

Supplementary Table S8: League table: Studies with sample size: ≥100. The values above the diet classes correspond to the difference in mean (95% CI) in HbA1c (%) between the row and columns (eg, the mean difference in average HbA1c between Low-Carb and Low-Fat diet is -0.11%)

HbA1c (%)								
Low-Carb	-0.37 (-0.86,0.11)	-0.17 (-0.89,0.54)	-0.37 (-0.78,0.03)	-0.09 (-0.60,0.42)	-0.47 (-0.82,-0.12)	-0.50 (-0.87,-0.12)	-0.57 (-0.87,-0.27)	-1.08 (-1.48,-0.67)
	Mediterranean	0.20 (-0.58,0.98)	0.00 (-0.51,0.52)	0.28 (-0.32,0.89)	-0.10 (-0.60,0.41)	-0.12 (-0.61,0.37)	-0.20 (-0.63,0.24)	-0.70 (-1.22,-0.19)
		Palaeolithic	-0.20 (-0.91,0.51)	0.08 (-0.70,0.86)	-0.30 (-1.01,0.41)	-0.33 (-1.02,0.36)	-0.40 (-1.05,0.25)	-0.91 (-1.61,-0.20)
			Vegetarian	0.28 (-0.23,0.79)	-0.10 (-0.49,0.29)	-0.13 (-0.49,0.24)	-0.20 (-0.48,0.08)	-0.70 (-1.09,-0.32)
				Low-GI/GL	-0.38 (-0.89,0.13)	-0.40 (-0.89,0.08)	-0.48 (-0.90,-0.05)	-0.98 (-1.49,-0.48)
					Moderate-Carb	-0.03 (-0.39,0.33)	-0.10 (-0.38,0.18)	-0.61 (-1.00,-0.21)
						High-Protein	-0.07 (-0.30,0.16)	-0.58 (-0.94,-0.22)
							Low-Fat	-0.51 (-0.78,-0.23)
								Control

Supplementary Table S9: League table: Studies with sample size: <100. The values above the diet classes correspond to the difference in mean (95% CI) in HbA1c (%) between the row and columns (eg, the mean difference in average HbA1c between Low-Carb and Low-Fat diet is -0.57%)

HbA1c (%)							
Low-Carb	-0.61 (-1.70,0.49)	-0.41 (-1.38,0.56)	-0.63 (-1.34,0.07)	-0.76 (-1.35,-0.17)	-0.82 (-1.57,-0.07)	-0.81 (-1.48,-0.14)	-1.23 (-1.97,-0.49)
	Mediterranean	0.20 (-0.91,1.31)	-0.03 (-0.94,0.89)	-0.15 (-1.10,0.79)	-0.21 (-1.14,0.71)	-0.20 (-1.06,0.66)	-0.62 (-1.55,0.30)
		Palaeolithic	-0.23 (-1.00,0.54)	-0.35 (-1.15,0.45)	-0.41 (-1.19,0.36)	-0.40 (-1.10,0.30)	-0.82 (-1.60,-0.05)
			Low GI/GL	-0.13 (-0.56,0.30)	-0.19 (-0.64,0.27)	-0.17 (-0.49,0.14)	-0.60 (-1.06,-0.14)
				Moderate-Carb	-0.06 (-0.57,0.45)	-0.05 (-0.43,0.34)	-0.47 (-0.99,0.05)
					High- Protein	0.01 (-0.32,0.35)	-0.41 (-0.88,0.06)
						Low-Fat	-0.42 (-0.75,-0.09)
							Control

Supplementary Table S10: League table: Studies mean age: ≥60 years of age. The values above the diet classes correspond to the difference in mean (95% CI) in HbA1c (%) between the row and columns (eg, the mean difference in average HbA1c between Low-Carb and Low-Fat diet is -0.81%)

HbA1c (%)							
Low Carb	0.18 (-0.07,0.43)	-0.00 (-0.34,0.33)	-0.06 (-0.30,0.18)	0.08 (-0.24,0.40)	-0.18 (-0.47,0.12)	-0.20 (-0.41,0.01)	-0.70 (-1.01,-0.39)
	Mediterranean	-0.18 (-0.48,0.11)	-0.24 (-0.40,-0.08)	-0.10 (-0.37,0.17)	-0.36 (-0.61,-0.10)	-0.38 (-0.52,-0.24)	-0.88 (-1.14,-0.62)
		Vegetarian	-0.06 (-0.34,0.22)	0.09 (-0.27,0.44)	-0.17 (-0.51,0.16)	-0.20 (-0.46,0.06)	-0.70 (-1.04,-0.35)
			Low-GI/GL	0.14 (-0.11,0.40)	-0.12 (-0.36,0.13)	-0.14 (-0.25,-0.04)	-0.64 (-0.88,-0.39)
				Moderate-Carb	-0.26 (-0.58,0.06)	-0.29 (-0.53,-0.05)	-0.78 (-1.08,-0.49)
					High-Protein	-0.03 (-0.24,0.18)	-0.52 (-0.83,-0.21)
						Low-Fat	-0.50 (-0.72,-0.27)
							Control

Supplementary Table S11: League table: Studies mean age: <60 years of age. The values above the diet classes correspond to the difference in mean (95% CI) in HbA1c (%) between the row and columns (eg, the mean difference in average HbA1c between Low-Carb and Low-Fat diet is -0.20%)

HbA1c (%)								
Low-Carb	-0.14 (-0.57,0.29)	-0.07 (-0.83,0.70)	-0.37 (-1.01,0.27)	-0.36 (-0.72,0.01)	-0.32 (-0.69,0.05)	-0.55 (-1.02,-0.08)	-0.47 (-0.78,-0.15)	-0.81 (-1.21,-0.41)
0.18 (-0.80,1.16)	Mediterranen	0.07 (-0.69,0.83)	-0.23 (-0.87,0.41)	-0.22 (-0.58,0.15)	-0.18 (-0.59,0.23)	-0.41 (-0.87,0.06)	-0.33 (-0.64,-0.02)	-0.67 (-1.06,-0.28)
0.13 (-1.54,1.81)	-0.05 (-1.70,1.61)	Palaeolithic	-0.30 (-1.19,0.59)	-0.29 (-1.01,0.44)	-0.25 (-1.00,0.50)	-0.48 (-1.26,0.30)	-0.40 (-1.10,0.30)	-0.74 (-1.48,-0.00)
-0.31 (-1.82,1.21)	-0.49 (-1.98,1.00)	-0.44 (-2.43,1.55)	Vegetarian	0.01 (-0.58,0.60)	0.05 (-0.57,0.67)	-0.18 (-0.83,0.48)	-0.10 (-0.66,0.46)	-0.44 (-1.05,0.17)
-0.33 (-1.16,0.50)	-0.51 (-1.38,0.37)	-0.46 (-2.05,1.13)	-0.02 (-1.44,1.40)	Low-GI/GL	0.04 (-0.28,0.36)	-0.19 (-0.59,0.21)	-0.11 (-0.31,0.08)	-0.46 (-0.77,-0.15)
0.12 (-0.80,1.03)	-0.06 (-1.04,0.91)	-0.02 (-1.66,1.63)	0.42 (-1.06,1.91)	0.45 (-0.36,1.25)	Moderate-Carb	-0.23 (-0.67,0.21)	-0.15 (-0.43,0.12)	-0.49 (-0.84,-0.15)
-0.57 (-2.00,0.87)	-0.75 (-2.16,0.66)	-0.70 (-2.63,1.23)	-0.26 (-2.05,1.53)	-0.24 (-1.57,1.09)	-0.68 (-2.09,0.72)	High-Protein	0.08 (-0.27,0.42)	-0.26 (-0.69,0.16)
-0.37 (-1.12,0.38)	-0.55 (-1.25,0.16)	-0.50 (-2.00,1.00)	-0.06 (-1.37,1.25)	-0.04 (-0.57,0.49)	-0.48 (-1.17,0.21)	0.20 (-1.02,1.42)	Low-Fat	-0.34 (-0.59,-0.10)
-1.07 (-2.04,-0.09)	-1.24 (-2.20,-0.29)	-1.20 (-2.83,0.43)	-0.76 (-2.22,0.71)	-0.74 (-1.57,0.09)	-1.18 (-2.05,-0.31)	-0.50 (-1.88,0.89)	-0.70 (-1.35,-0.05)	Control
Fasting glucose (mmol/l)								

Table S12: League table: Low risk of bias studies. The values above the diet classes correspond to the difference in mean (95% CI) in HbA1c (%) between the row and columns (eg, the mean difference in average HbA1c between Low-Carb and Low-Fat diet is -0.47%) The value below the diet classes correspond to the

difference in mean in fasting glucose (mmol/l) between the column and the row (eg, the mean difference in average fasting glucose between Low-Carb and Low-Fat diet is -0.37 mmol/l)

HbA1c (%)								
Low-Carb	-0.08 (-0.41,0.25)	-0.01 (-0.72,0.71)	-0.21 (-0.60,0.19)	-0.29 (-0.58,0.01)	-0.29 (-0.58,0.01)	-0.39 (-0.72,-0.07)	-0.41 (-0.66,-0.15)	-0.41 (-0.66,-0.15)
0.33 (-0.42,1.07)	Mediterranen	0.07 (-0.63,0.78)	-0.13 (-0.50,0.24)	-0.21 (-0.48,0.06)	-0.21 (-0.50,0.09)	-0.32 (-0.62,-0.01)	-0.33 (-0.55,-0.11)	-0.80 (-1.08,-0.52)
0.21 (-1.20,1.63)	-0.11 (-1.45,1.23)	Palaeolithic	-0.20 (-0.93,0.53)	-0.28 (-0.97,0.41)	-0.28 (-0.98,0.42)	-0.39 (-1.09,0.31)	-0.40 (-1.07,0.27)	-0.87 (-1.57,-0.18)
0.01 (-0.90,0.91)	-0.32 (-1.10,0.46)	-0.21 (-1.63,1.22)	Vegetarian	-0.08 (-0.42,0.26)	-0.08 (-0.44,0.28)	-0.19 (-0.55,0.18)	-0.20 (-0.50,0.10)	-0.67 (-1.03,-0.32)
-0.28 (-0.96,0.40)	-0.60 (-1.17,-0.04)	-0.49 (-1.82,0.83)	-0.29 (-1.04,0.46)	Low-GI/GL	0.00 (-0.25,0.25)	-0.11 (-0.37,0.15)	-0.12 (-0.28,0.04)	-0.59 (-0.85,-0.34)
-0.08 (-0.77,0.61)	-0.41 (-0.96,0.15)	-0.29 (-1.61,1.03)	-0.09 (-0.83,0.65)	0.20 (-0.30,0.70)	Moderate-Carb	-0.11 (-0.39,0.18)	-0.12 (-0.32,0.08)	-0.59 (-0.86,-0.33)
-0.20 (-0.98,0.58)	-0.53 (-1.15,0.10)	-0.42 (-1.76,0.93)	-0.21 (-1.00,0.58)	0.08 (-0.52,0.67)	-0.12 (-0.70,0.46)	High-Protein	-0.01 (-0.22,0.19)	-0.49 (-0.77,-0.20)
-0.29 (-0.92,0.35)	-0.61 (-1.04,-0.18)	-0.50 (-1.77,0.77)	-0.29 (-0.94,0.36)	-0.01 (-0.38,0.37)	-0.21 (-0.57,0.16)	-0.08 (-0.54,0.37)	Low-Fat	-0.47 (-0.67,-0.28)
-1.28 (-2.01,-0.55)	-1.61 (-2.17,-1.04)	-1.50 (-2.82,-0.17)	-1.29 (-2.04,-0.54)	-1.00 (-1.53,-0.48)	-1.20 (-1.70,-0.70)	-1.08 (-1.67,-0.49)	-1.00 (-1.36,-0.63)	Control
Fasting glucose (mmol/l)								

Table S13: League table: High risk of bias trials excluded. The values above the diet classes correspond to the difference in mean (95% CI) in HbA1c (%) between the row and columns (eg, the mean difference in average HbA1c between Low-Carb and Low-Fat diet is -0.41%) The value below the diet classes

correspond to the difference in mean in fasting glucose (mmol/l) between the column and the row (eg, the mean difference in average fasting glucose between Low-Carb and Low-Fat diet is -0.29 mmol/l)

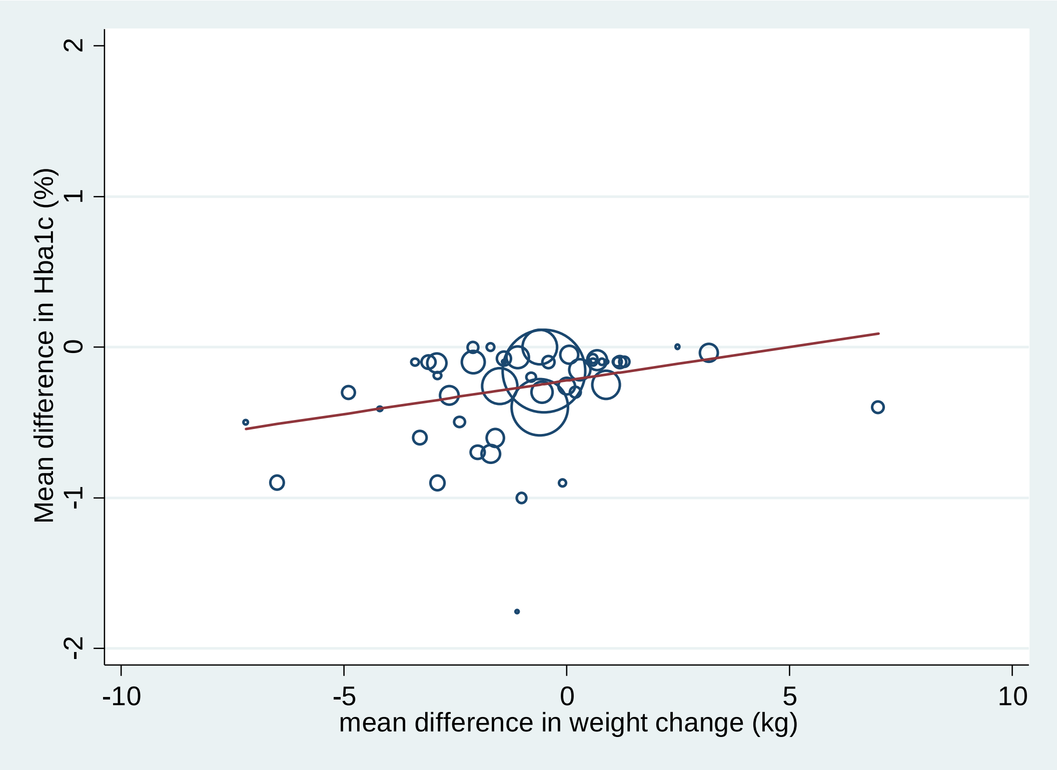

Supplementary Figure S13: Meta-regression showing the association between mean differences in HbA1c (%) and mean difference in weight change between dietary approaches

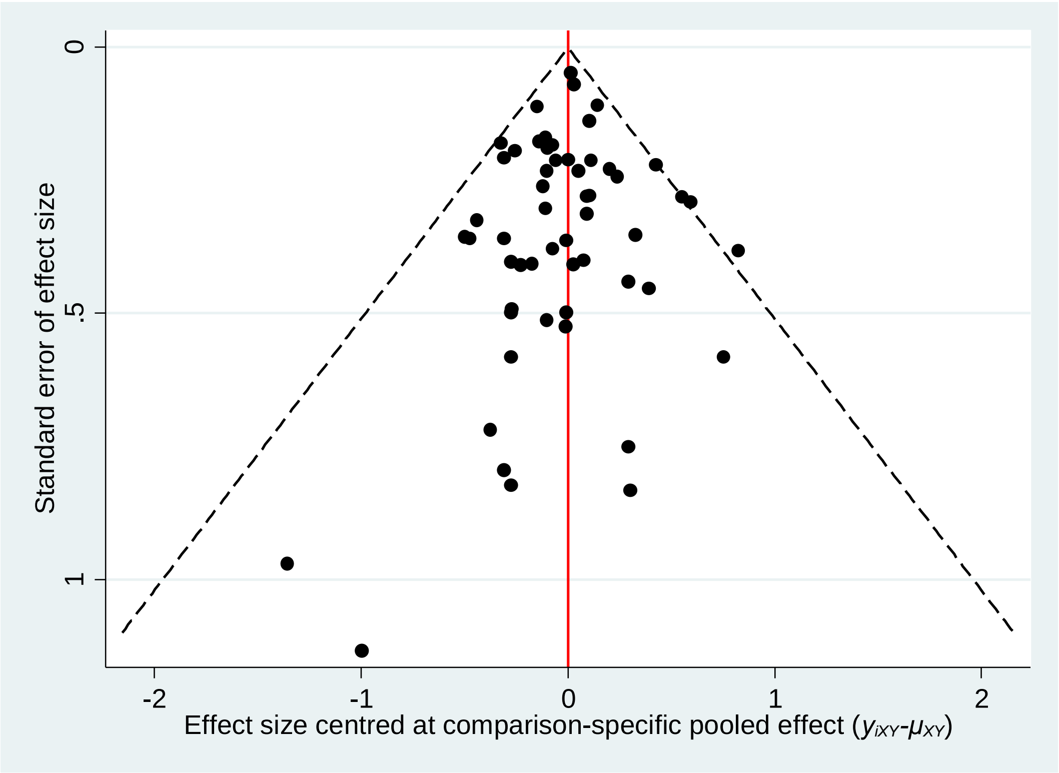

Supplementary Figure S14: Comparison-adjusted funnel plot for HbA1c involving all studies comparing all low-fat diets vs. all other dietary approaches.

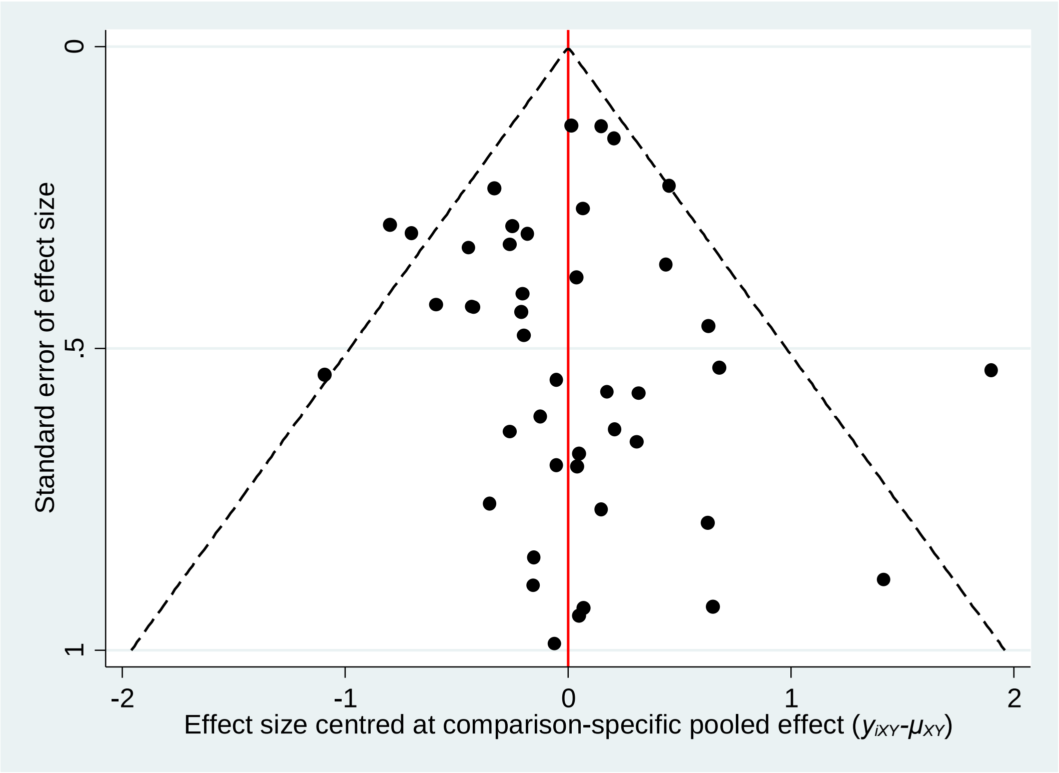

Supplementary Figure S15: Comparison-adjusted funnel plot for fasting glucose involving all studies comparing a low fat diet vs. all other approaches

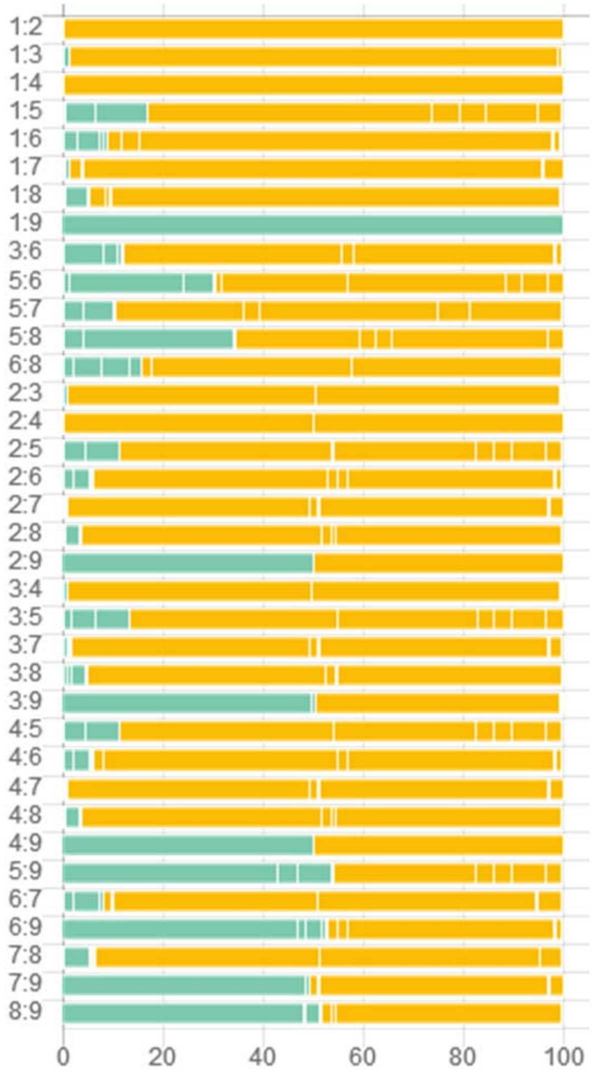

Supplementary Figure S16: Bar-graph showing for every comparison the percentage of information coming from low (green), moderate (yellow) and high (red) risk of bias studies [1=Low-fat, 2=Vegetarian, 3=Mediterranean, 4=High-Protein, 5=Moderate-carbohydrate, 6=Low-carbohydrate, 7=control, 8=Low GI/GL, 9=Palaeolithic].

Supplementary Appendix S2:

We evaluated the credibility of the evidence from network meta-analysis based on the approach suggested by Salanti G et al. Evaluating the quality of evidence from a network meta-analysis. PloS One. 2014;9(7):e99682 that extends the GRADE system into network meta-analysis.

To evaluate study limitations we used the online tool CiNeMA available from http://ec2-52-28-232-32.eu-central-1.compute.amazonaws.com:8004/ocpu/library/contribution/www/ that combines the contribution of each direct comparison to the estimation (see Chaimani et al. Graphical tools for network meta-analysis in Stata. Plos One 2013 and Krahn et al. A graphical tool for locating inconsistency in network meta-analyses. BMC Med Res Methodol 2013) with the risk of bias assessments for each direct comparison and produces a bar-graph (Supplementary Figure 16). The bar-graph shows for the network estimate of every pairwise comparison how much information comes from low (green), moderate (yellow) or high (red) risk of bias studies.

Below we present our GRADE evaluation for the primary outcome and all comparisons between the different dietary approaches.

Comparison	Risk of bias	Indirectness	Inconsistency	Imprecision	Publications bias	Rating
1:2	Serious concerns	Serious concerns	No serious concerns	Serious concerns	Serious concerns	Low
1:3	Serious concerns	Serious concerns	Very serious concerns	No serious concerns	Serious concerns	Very low
1:4	Serious concerns	Serious concerns	No serious concerns	Serious concerns	Serious concerns	Low
1:5	Serious concerns	Serious concerns	Serious concerns	Serious concerns	Serious concerns	Low
1:6	Serious concerns	Serious concerns	Very serious concerns	No serious concerns	Serious concerns	Very low
1:7	Serious concerns	Serious concerns	Serious concerns	No serious concerns	Serious concerns	Low
1:8	Serious concerns	Serious concerns	Very serious concerns	Serious concerns	Serious concerns	Very low
1:9	No serious concerns	Serious concerns	No serious concerns	Serious concerns	Serious concerns	Moderate
3:6	Serious concerns	Serious concerns	Serious concerns	Serious concerns	Serious concerns	Low
5:6	Serious concerns	Serious concerns	Very serious concerns	Serious concerns	Serious concerns	Very low
5:7	Serious concerns	Serious concerns	Serious concerns	No serious concerns	Serious concerns	Low
5:8	Serious concerns	Serious concerns	Serious concerns	Serious concerns	Serious concerns	Low
6:8	Serious concerns	Serious concerns	Serious concerns	Serious concerns	Serious concerns	Low
2:3	Serious concerns	Serious concerns	Serious concerns	Serious concerns	Serious concerns	Low
2:4	Serious concerns	Serious concerns	Serious concerns	Serious concerns	Serious concerns	Low
2:5	Serious concerns	Serious concerns	Serious concerns	Serious concerns	Serious concerns	Low
2:6	Serious concerns	Serious concerns	Serious concerns	Serious concerns	Serious concerns	Low

2:7	Serious concerns	Serious concerns	Serious concerns	No serious concerns	Serious concerns	Low
2:8	Serious concerns	Serious concerns	Serious concerns	Serious concerns	Serious concerns	Low
2:9	Serious concerns	Serious concerns	Serious concerns	Serious concerns	Serious concerns	Low
3:4	Serious concerns	Serious concerns	Very serious concerns	No serious concerns	Serious concerns	Very low
3:5	Serious concerns	Serious concerns	Serious concerns	Serious concerns	Serious concerns	Low
3:7	Serious concerns	Serious concerns	Serious concerns	No serious concerns	Serious concerns	Low
3:8	Serious concerns	Serious concerns	Serious concerns	No serious concerns	Serious concerns	Low
3:9	No serious concerns	Serious concerns	Serious concerns	Serious concerns	Serious concerns	Moderate
4:5	Serious concerns	Serious concerns	Serious concerns	Serious concerns	Serious concerns	Low
4:6	Serious concerns	Serious concerns	Very serious concerns	Serious concerns	Serious concerns	Very low
4:7	Serious concerns	Serious concerns	Serious concerns	No serious concerns	Serious concerns	Low
4:8	Serious concerns	Serious concerns	Serious concerns	Serious concerns	Serious concerns	Low
4:9	Serious concerns	Serious concerns	Serious concerns	Serious concerns	Serious concerns	Low
5:9	No serious concerns	Serious concerns	Serious concerns	Serious concerns	Serious concerns	Moderate
6:7	Serious concerns	Serious concerns	Serious concerns	Serious concerns	Serious concerns	Low
6:9	No serious concerns	Serious concerns	Serious concerns	Serious concerns	Serious concerns	Moderate
7:8	Serious concerns	Serious concerns	Serious concerns	No serious concerns	Serious concerns	Low
8:9	Serious concerns	Serious concerns	Serious concerns	No serious concerns	Serious concerns	Low
8:9	No serious concerns	Serious concerns	Serious concerns	Serious concerns	Serious concerns	Moderate

[1=Low-fat, 2=Vegetarian, 3=Mediterranean, 4=High-Protein, 5=Moderate-carbohydrate, 6=Low-carbohydrate, 7=control, 8=Low GI/GL, 9=Palaeolithic].

Supplementary References of excluded studies

1. Argiana V, Kanellos P, Makrilakis K, et al. The effect of consumption of low-glycemic-index and low-glycemic-load desserts on anthropometric parameters and inflammatory markers in patients with type 2 diabetes mellitus. Eur J Nutr. 2015;54(7):1173-80. doi:10.1007/s00394-014-0795-8
2. Balfego M, Canivell S, Hanzu FA, et al. Effects of sardine-enriched diet on metabolic control, inflammation and gut microbiota in drug-naive patients with type 2 diabetes: a pilot randomized trial. Lipids Health Dis. 2016;15:78. doi:10.1186/s12944-016-0245-0
3. Fuller NR, Caterson ID, Sainsbury A, et al. The effect of a high-egg diet on cardiovascular risk factors in people with type 2 diabetes: the Diabetes and Egg (DIABEGG) study-a 3-mo randomized controlled trial. Am J Clin Nutr. 2015;101(4):705-13. doi:10.3945/ajcn.114.096925
4. Heilbronn LK, Noakes M, Clifton PM. The effect of high- and low-glycemic index energy restricted diets on plasma lipid and glucose profiles in type 2 diabetic subjects with varying glycemic control. J Am Coll Nutr. 2002;21(2):120-7.
5. Barnard ND, Cohen J, Jenkins DJ, et al. A low-fat vegan diet improves glycemic control and cardiovascular risk factors in a randomized clinical trial in individuals with type 2 diabetes. Diabetes Care. 2006;29(8):1777-83. doi:10.2337/dc06-0606
6. Brekke HK, Jansson PA, Lenner RA. Long-term (1- and 2-year) effects of lifestyle intervention in type 2 diabetes relatives. Diabetes research and clinical practice. 2005;70(3):225-34. doi:10.1016/j.diabres.2005.03.027
7. Brinkworth GD, Luscombe-Marsh ND, Thompson CH, et al. Long-term effects of very low-carbohydrate and high-carbohydrate weight-loss diets on psychological health in obese adults with type 2 diabetes: randomized controlled trial. Journal of internal medicine. 2016;280(4):388-97. doi:10.1111/joim.12501
8. Cohen AE, Johnston CS. Almond ingestion at mealtime reduces postprandial glycemia and chronic ingestion reduces hemoglobin A(1c) in individuals with well-controlled type 2 diabetes mellitus. Metabolism. 2011;60(9):1312-7. doi:10.1016/j.metabol.2011.01.017
9. Ferdowsian HR, Barnard ND, Hoover VJ, et al. A multicomponent intervention reduces body weight and cardiovascular risk at a GEICO corporate site. American journal of health promotion : AJHP. 2010;24(6):384-7. doi:10.4278/ajhp.081027-QUAN-255
10. Garg A, Bantle JP, Henry RR, et al. Effects of varying carbohydrate content of diet in patients with non-insulin-dependent diabetes mellitus. Jama. 1994;271(18):1421-8.
11. Gutierrez M, Akhavan M, Jovanovic L, Peterson CM. Utility of a short-term 25% carbohydrate diet on improving glycemic control in type 2 diabetes mellitus. J Am Coll Nutr. 1998;17(6):595-600.
12. Haimoto H, Iwata M, Wakai K, Umegaki H. Long-term effects of a diet loosely restricting carbohydrates on HbA1c levels, BMI and tapering of sulfonylureas in type 2 diabetes: a 2-year follow-up study. Diabetes research and clinical practice. 2008;79(2):350-6. doi:10.1016/j.diabres.2007.09.009
13. Hussain TA, Mathew TC, Dashti AA, Asfar S, Al-Zaid N, Dashti HM. Effect of low-calorie versus low-carbohydrate ketogenic diet in type 2 diabetes. Nutrition (Burbank, Los Angeles County, Calif.). 2012;28(10):1016-21. doi:10.1016/j.nut.2012.01.016
14. Jenkins DJ, Kendall CW, Augustin LS, et al. Effect of wheat bran on glycemic control and risk factors for cardiovascular disease in type 2 diabetes. Diabetes Care. 2002;25(9):1522-8.
15. Kim HH, Kim YJ, Lee SY, et al. Interactive effects of an isocaloric high-protein diet and resistance exercise on body composition, ghrelin, and metabolic and hormonal parameters in untrained young men: A randomized clinical trial. Journal of diabetes investigation. 2014;5(2):242-7. doi:10.1111/jdi.12148
16. Lalor BC, Bhatnagar D, Winocour PH, et al. Placebo-controlled trial of the effects of guar gum and metformin on fasting blood glucose and serum lipids in obese, type 2 diabetic patients. Diabetic medicine : a journal of the British Diabetic Association. 1990;7(3):242-5.
17. Mayer SB, Jeffreys AS, Olsen MK, McDuffie JR, Feinglos MN, Yancy WS, Jr. Two diets with different haemoglobin A1c and antiglycaemic medication effects despite similar weight loss in type 2 diabetes. Diabetes Obes Metab. 2014;16(1):90-3. doi:10.1111/dom.12191

18. Li SC, Liu YH, Liu JF, Chang WH, Chen CM, Chen CY. Almond consumption improved glycemic control and lipid profiles in patients with type 2 diabetes mellitus. Metabolism. 2011;60(4):474-9. doi:10.1016/j.metabol.2010.04.009
19. Ma Y, Njike VY, Millet J, et al. Effects of walnut consumption on endothelial function in type 2 diabetic subjects: a randomized controlled crossover trial. Diabetes Care. 2010;33(2):227-32. doi:10.2337/dc09-1156
20. Nielsen JV, Jonsson E, Nilsson AK. Lasting improvement of hyperglycaemia and bodyweight: low-carbohydrate diet in type 2 diabetes. A brief report. Upsala journal of medical sciences. 2005;110(2):179-83.
21. Paisey RB, Frost J, Harvey P, et al. Five year results of a prospective very low calorie diet or conventional weight loss programme in type 2 diabetes. Journal of human nutrition and dietetics : the official journal of the British Dietetic Association. 2002;15(2):121-7.
22. Pittas AG, Roberts SB, Das SK, et al. The effects of the dietary glycemic load on type 2 diabetes risk factors during weight loss. Obesity (Silver Spring, Md.). 2006;14(12):2200-9. doi:10.1038/oby.2006.258
23. Tapsell LC, Gillen LJ, Patch CS, et al. Including walnuts in a low-fat/modified-fat diet improves HDL cholesterol-to-total cholesterol ratios in patients with type 2 diabetes. Diabetes Care. 2004;27(12):2777-83.
24. Tapsell LC, Batterham MJ, Teuss G, et al. Long-term effects of increased dietary polyunsaturated fat from walnuts on metabolic parameters in type II diabetes. Eur J Clin Nutr. 2009;63(8):1008-15. doi:10.1038/ejcn.2009.19
25. Tay J, Luscombe-Marsh ND, Thompson CH, et al. A very low-carbohydrate, low-saturated fat diet for type 2 diabetes management: a randomized trial. Diabetes Care. 2014;37(11):2909-18. doi:10.2337/dc14-0845
26. Toobert DJ, Glasgow RE, Strycker LA, Barrera M, Jr., Ritzwoller DP, Weidner G. Long-term effects of the Mediterranean lifestyle program: a randomized clinical trial for postmenopausal women with type 2 diabetes. The international journal of behavioral nutrition and physical activity. 2007;4:1. doi:10.1186/1479-5868-4-1
27. Uusitupa M, Siitonen O, Savolainen K, Silvasti M, Penttila I, Parviainen M. Metabolic and nutritional effects of long-term use of guar gum in the treatment of noninsulin-dependent diabetes of poor metabolic control. Am J Clin Nutr. 1989;49(2):345-51.
28. Wien M, Oda K, Sabate J. A randomized controlled trial to evaluate the effect of incorporating peanuts into an American Diabetes Association meal plan on the nutrient profile of the total diet and cardiometabolic parameters of adults with type 2 diabetes. Nutr J. 2014;13:10. doi:10.1186/1475-2891-13-10
29. Wing RR, Marcus MD, Salata R, Epstein LH, Miaskiewicz S, Blair EH. Effects of a very-low-calorie diet on long-term glycemic control in obese type 2 diabetic subjects. Arch Intern Med. 1991;151(7):1334-40.
30. Wing RR, Blair E, Marcus M, Epstein LH, Harvey J. Year-long weight loss treatment for obese patients with type II diabetes: does including an intermittent very-low-calorie diet improve outcome? Am J Med. 1994;97(4):354-62.
31. Wycherley TP, Thompson CH, Buckley JD, et al. Long-term effects of weight loss with a very-low carbohydrate, low saturated fat diet on flow mediated dilatation in patients with type 2 diabetes: A randomised controlled trial. Atherosclerosis. 2016;252:28-31. doi:10.1016/j.atherosclerosis.2016.07.908
32. Yancy WS, Jr., Foy M, Chalecki AM, Vernon MC, Westman EC. A low-carbohydrate, ketogenic diet to treat type 2 diabetes. Nutrition & metabolism. 2005;2:34. doi:10.1186/1743-7075-2-34
33. Pearce KL, Clifton PM, Noakes M. Egg consumption as part of an energy-restricted high-protein diet improves blood lipid and blood glucose profiles in individuals with type 2 diabetes. Br J Nutr. 2011;105(4):584-92. doi:10.1017/s0007114510003983
34. Parham M, Heidari S, Khorramirad A, et al. Effects of pistachio nut supplementation on blood glucose in patients with type 2 diabetes: a randomized crossover trial. The review of diabetic studies : RDS. 2014;11(2):190-6. doi:10.1900/rds.2014.11.190
35. Hakala P, Karvetti RL. Weight reduction on lactovegetarian and mixed diets. Changes in weight, nutrient intake, skinfold thicknesses and blood pressure. Eur J Clin Nutr. 1989;43(6):421-30.

36. Fontes-Villalba M, Lindeberg S, Granfeldt Y, et al. Palaeolithic diet decreases fasting plasma leptin concentrations more than a diabetes diet in patients with type 2 diabetes: a randomised cross-over trial. Cardiovascular diabetology. 2016;15:80. doi:10.1186/s12933-016-0398-1
37. Frost G, Wilding J, Beecham J. Dietary advice based on the glycaemic index improves dietary profile and metabolic control in type 2 diabetic patients. Diabetic medicine : a journal of the British Diabetic Association. 1994;11(4):397-401.
38. Mancini JG, Filion KB, Atallah R, Eisenberg MJ. Systematic Review of the Mediterranean Diet for Long-Term Weight Loss. Am J Med. 2016;129(4):407-15.e4. doi:10.1016/j.amjmed.2015.11.028
39. Ajala O, English P, Pinkney J. Systematic review and meta-analysis of different dietary approaches to the management of type 2 diabetes. Am J Clin Nutr. 2013;97(3):505-16. doi:10.3945/ajcn.112.042457
40. Carter P, Achana F, Troughton J, Gray LJ, Khunti K, Davies MJ. A Mediterranean diet improves HbA1c but not fasting blood glucose compared to alternative dietary strategies: a network meta-analysis. Journal of human nutrition and dietetics : the official journal of the British Dietetic Association. 2014;27(3):280-97. doi:10.1111/jhn.12138
41. Brand-Miller J, Hayne S, Petocz P, Colagiuri S. Low-glycemic index diets in the management of diabetes: a meta-analysis of randomized controlled trials. Diabetes Care. 2003;26(8):2261-7.
42. Chen L, Pei JH, Kuang J, et al. Effect of lifestyle intervention in patients with type 2 diabetes: a meta-analysis. Metabolism. 2015;64(2):338-47. doi:10.1016/j.metabol.2014.10.018
43. Emadian A, Andrews RC, England CY, Wallace V, Thompson JL. The effect of macronutrients on glycaemic control: a systematic review of dietary randomised controlled trials in overweight and obese adults with type 2 diabetes in which there was no difference in weight loss between treatment groups. Br J Nutr. 2015;114(10):1656-66. doi:10.1017/s0007114515003475
44. Feinman RD, Pogozelski WK, Astrup A, et al. Dietary carbohydrate restriction as the first approach in diabetes management: critical review and evidence base. Nutrition (Burbank, Los Angeles County, Calif.). 2015;31(1):1-13. doi:10.1016/j.nut.2014.06.011
45. Franz MJ, Boucher JL, Rutten-Ramos S, VanWormer JJ. Lifestyle weight-loss intervention outcomes in overweight and obese adults with type 2 diabetes: a systematic review and meta-analysis of randomized clinical trials. J Acad Nutr Diet. 2015;115(9):1447-63. doi:10.1016/j.jand.2015.02.031
46. Garg A. High-monounsaturated-fat diets for patients with diabetes mellitus: a meta-analysis. Am J Clin Nutr. 1998;67(3 Suppl):577s-82s.
47. Huang XL, Pan JH, Chen D, Chen J, Chen F, Hu TT. Efficacy of lifestyle interventions in patients with type 2 diabetes: A systematic review and meta-analysis. European journal of internal medicine. 2016;27:37-47. doi:10.1016/j.ejim.2015.11.016
48. Huo R, Du T, Xu Y, et al. Effects of Mediterranean-style diet on glycemic control, weight loss and cardiovascular risk factors among type 2 diabetes individuals: a meta-analysis. Eur J Clin Nutr. 2015;69(11):1200-8. doi:10.1038/ejcn.2014.243
49. Dong JY, Zhang ZL, Wang PY, Qin LQ. Effects of high-protein diets on body weight, glycaemic control, blood lipids and blood pressure in type 2 diabetes: meta-analysis of randomised controlled trials. Br J Nutr. 2013;110(5):781-9. doi:10.1017/s0007114513002055
50. Kirk JK, Graves DE, Craven TE, Lipkin EW, Austin M, Margolis KL. Restricted-carbohydrate diets in patients with type 2 diabetes: a meta-analysis. J Am Diet Assoc. 2008;108(1):91-100. doi:10.1016/j.jada.2007.10.003
51. Post RE, Mainous AG, 3rd, King DE, Simpson KN. Dietary fiber for the treatment of type 2 diabetes mellitus: a meta-analysis. Journal of the American Board of Family Medicine : JABFM. 2012;25(1):16-23. doi:10.3122/jabfm.2012.01.110148
52. Schellenberg ES, Dryden DM, Vandermeer B, Ha C, Korownyk C. Lifestyle interventions for patients with and at risk for type 2 diabetes: a systematic review and meta-analysis. Ann Intern Med. 2013;159(8):543-51. doi:10.7326/0003-4819-159-8-201310150-00007
53. Schwingshackl L, Hoffmann G. Comparison of the long-term effects of high-fat v. low-fat diet consumption on cardiometabolic risk factors in subjects with abnormal glucose metabolism: a systematic review and meta-analysis. Br J Nutr. 2014;111(12):2047-58. doi:10.1017/s0007114514000464

54. Schwingshackl L, Hoffmann G. Mediterranean dietary pattern, inflammation and endothelial function: a systematic review and meta-analysis of intervention trials. Nutr Metab Cardiovasc Dis. 2014;24(9):929-39. doi:10.1016/j.numecd.2014.03.003
55. Shirani F, Salehi-Abargouei A, Azadbakht L. Effects of Dietary Approaches to Stop Hypertension (DASH) diet on some risk for developing type 2 diabetes: a systematic review and meta-analysis on controlled clinical trials. Nutrition (Burbank, Los Angeles County, Calif.). 2013;29(7-8):939-47. doi:10.1016/j.nut.2012.12.021
56. Terranova CO, Brakenridge CL, Lawler SP, Eakin EG, Reeves MM. Effectiveness of lifestyle-based weight loss interventions for adults with type 2 diabetes: a systematic review and meta-analysis. Diabetes Obes Metab. 2015;17(4):371-8. doi:10.1111/dom.12430
57. Tobias DK, Chen M, Manson JE, Ludwig DS, Willett W, Hu FB. Effect of low-fat diet interventions versus other diet interventions on long-term weight change in adults: a systematic review and meta-analysis. Lancet Diabetes Endocrinol. 2015;3(12):968-79. doi:10.1016/s2213-8587(15)00367-8
58. Viguiliouk E, Kendall CW, Blanco Mejia S, et al. Effect of tree nuts on glycemic control in diabetes: a systematic review and meta-analysis of randomized controlled dietary trials. PLoS One. 2014;9(7):e103376. doi:10.1371/journal.pone.0103376
59. Yokoyama Y, Barnard ND, Levin SM, Watanabe M. Vegetarian diets and glycemic control in diabetes: a systematic review and meta-analysis. Cardiovascular diagnosis and therapy. 2014;4(5):373-82. doi:10.3978/j.issn.2223-3652.2014.10.04
